# SCRaMbLE generates evolved yeasts with increased alkali tolerance

**DOI:** 10.1186/s12934-019-1102-4

**Published:** 2019-03-11

**Authors:** Lu Ma, Yunxiang Li, Xinyu Chen, Mingzhu Ding, Yi Wu, Ying-Jin Yuan

**Affiliations:** 1 Frontier Science Center for Synthetic Biology and Key Laboratory of Systems Bioengineering (Ministry of Education), Tianjin University, Tianjin, 300072 China; 2 Collaborative Innovation Center of Chemical Science and Engineering (Tianjin), School of Chemical Engineering and Technology, Tianjin University, Tianjin, 300072 China

**Keywords:** SCRaMbLE, Alkali tolerance, Synthetic biology, *Saccharomyces cerevisiae*, *SPT2*

## Abstract

**Background:**

Strains with increased alkali tolerance have a broad application in industrial, especially for bioremediation, biodegradation, biocontrol and production of bio-based chemicals. A novel synthetic chromosome recombination and modification by LoxP-mediated evolution (SCRaMbLE) system has been introduced in the synthetic yeast genome (Sc 2.0), which enables generation of a yeast library with massive structural variations and potentially drives phenotypic evolution. The structural variations including deletion, inversion and duplication have been detected within synthetic yeast chromosomes.

**Results:**

Haploid yeast strains harboring either one (synV) or two (synV and synX) synthetic chromosomes were subjected to SCRaMbLE. Seven of evolved strains with increased alkali tolerance at pH 8.0 were generated through multiple independent SCRaMbLE experiments. Various of structural variations were detected in evolved yeast strains by PCRTag analysis and whole genome sequencing including two complex structural variations. One possessed an inversion of 20,743 base pairs within which YEL060C (*PRB1*) was deleted simultaneously, while another contained a duplication region of 9091 base pairs in length with a deletion aside. Moreover, a common deletion region with length of 11,448 base pairs was mapped in four of the alkali-tolerant strains. We further validated that the deletion of YER161C (*SPT2*) within the deleted region could increase alkali tolerance in *Saccharomyces cerevisiae*.

**Conclusions:**

SCRaMbLE system provides a simple and efficient way to generate evolved yeast strains with enhanced alkali tolerance. Deletion of YER161C (*SPT2*) mapped by SCRaMbLE can improve alkali tolerance in *S. cerevisiae*. This study enriches our understanding of alkali tolerance in yeast and provides a standard workflow for the application of SCRaMbLE system to generate various phenotypes that may be interesting for industry and extend understanding of phenotype-genotype relationship.

**Electronic supplementary material:**

The online version of this article (10.1186/s12934-019-1102-4) contains supplementary material, which is available to authorized users.

## Background

Genomic variation drives phenotypic diversification in populations and evolutionary changes among different species [1–3]. Genome evolution in nature is a long-term process with accumulation of DNA variation that mainly involves single nucleotide polymorphisms (SNP), insertion-deletion (InDel) and structural variation (SV) [4–6]. Accordingly, techniques of manipulating varied length of DNA are constantly being developed and updated to engineer biological systems. Traditional techniques such as physical and chemical mutagenesis and error-prone PCR, could generate mutations in vivo and in vitro at the level of single nucleotide polymorphisms or insertion-deletion [7, 8]. Genome-editing technique mediated by TALEN, ZFN or CRISPR-Cas9 has made it possible to edit one or multiple targets on the genome rationally and precisely [9–11]. However, very few techniques related to large-scale genome rearrangement are reported to generate a structural variation library. The synthetic yeast genome project (Sc 2.0 project) is designed to encode an inducible genome rearrangement system in the chemically synthesized yeast genome by thousands of loxPsym sites inserted in the 3′ untranslated region (UTR) of nonessential genes [12–18]. With the expression of Cre recombinase in vivo, the synthetic chromosome recombination and modification by LoxP-mediated evolution (SCRaMbLE) system could drive generation of a yeast library with massive structural variations. The structural variations including deletion, inversion and duplication have been detected within synthetic yeast chromosomes and ring synthetic yeast chromosomes after SCRaMbLEing [19, 20]. Moreover, some evolved yeast strains with enhanced phenotype (i.e., temperature tolerance, caffeine tolerance, production of carotenoids) have been generated by SCRaMbLE system, showing promising application of this technology [21–27].

**Fig. 1:**
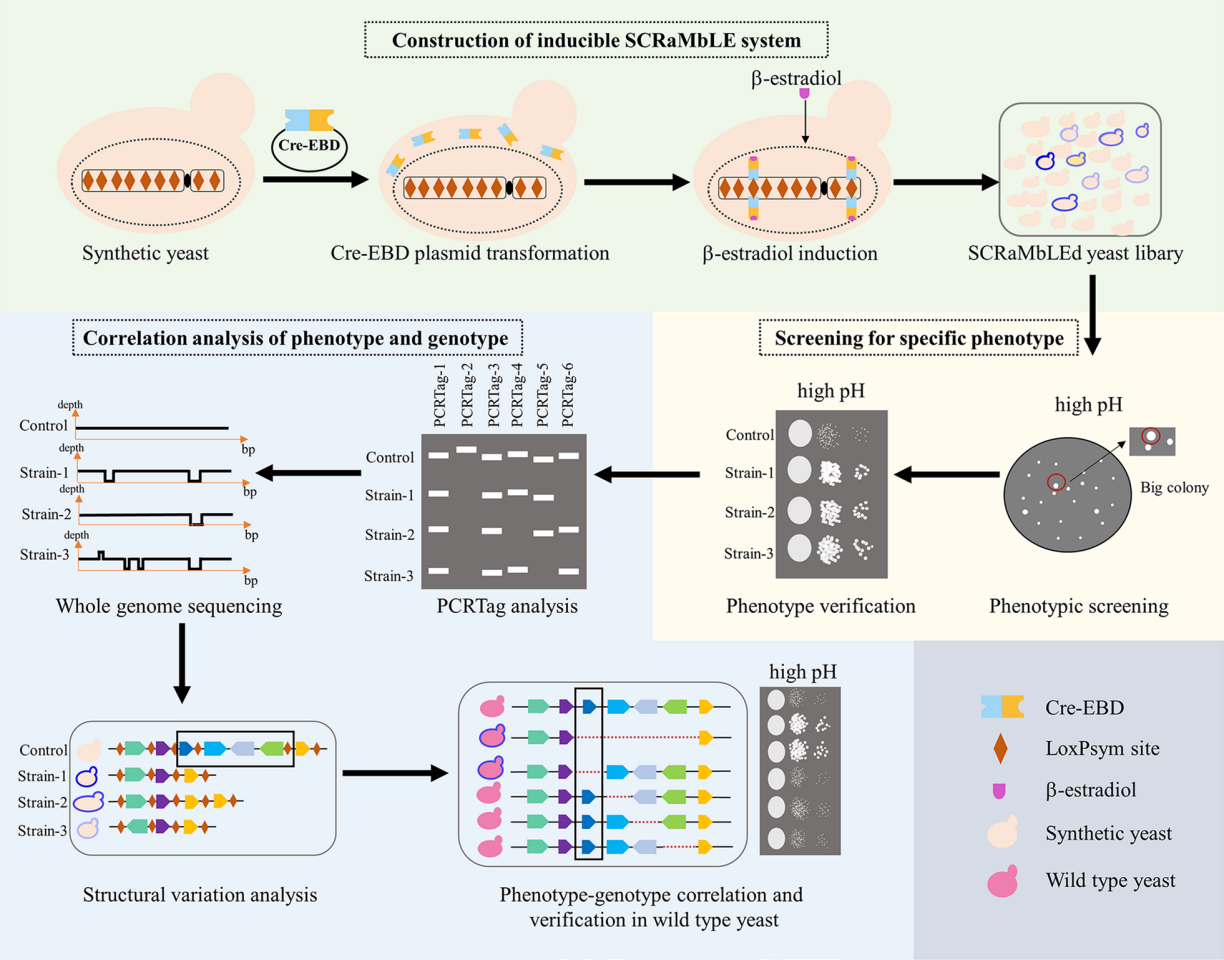
The workflow of using SCRaMbLE to improve and analyze alkali tolerance in yeast. Three sections in varied colors indicated three sequential steps of the whole process including construction of inducible SCRaMbLE system, screening for specific phenotype, correlation analysis of phenotype and genotype

Extracellular pH, as an important environmental condition, has a significant influence on the survival and growth of cells. As a model organism, *S. cerevisiae* grows more rapidly in acidic than neutral or alkaline pH [28, 29]. It is important to study this underling mechanism for the application of industrial fermentation in alkaline environments. The discoveries of genes related to alkali tolerance in previous studies have been reported via random mutation, long-term adaptive evolution, deletion or overexpression of a dubious gene [29–32]. The transcriptional response to alkaline stress in *S. cerevisiae* was also studied by a short-term exposure in alkaline pH [33]. However, these methods are non-rational designed and make it complicated to uncover phenotype-genotype correlations. In this study, we accelerated the evolution of synthetic yeast strains in alkaline pH using SCRaMbLE system. Two synthetic yeast strains synV and synV&X, which harbor one synthetic yeast chromosome V and two synthetic yeast chromosomes V and X respectively, were used as initial strains [12, 13]. Through multiple independent SCRaMbLE experiments, seven of evolved strains with increased alkali tolerance at pH 8.0 were obtained. Many structural variations were detected in the evolved yeast strains by PCRTag analysis and whole genome sequencing. A common deletion region with length of 11,448 base pairs has been mapped in four of the alkali-tolerant strains. We further validated that the deletion of YER161C (*SPT2*) in this region could lead to the enhanced phenotype under alkaline stress. The SCRaMbLE method provides an efficient way to generate evolved strains with increased alkali tolerance and a straightforward way to dissect the correlation of phenotype and genotype (Fig. 1).

## Results and discussion

### Construction and characterization of SCRaMbLE system

Synthetic yeast chromosome V (synV) is 536,024 base pairs long encoding 176 loxPsym sites [13]. Synthetic yeast chromosome X (synX) is 707,459 base pairs in length encoding 245 loxPsym sites [12]. Haploid strains bearing one (synV) or two (synV and synX) synthetic chromosomes were subjected to SCRaMbLE in this study. Cre recombinase fused with estrogen-binding domain (EBD) has been used to control the SCRaMbLE system in previous studies [19, 22]. Two Cre-EBD plasmids pYW079 (pRS416-pSCW11-Cre-EBD) and pYW180 (pRS416-pCLB2-Cre-EBD) with different promoters were transformed to strains yXZX846 (synV) and yYW169 (synV&X). pCLB2 is a G2/M-specific promoter which could lead cells to expose to Cre recombinase continually in each cell cycle [34], while pSCW11 is a strong promoter which only initiates transcription in daughter cells [35]. β-Estradiol was added in medium to enable the fusion protein Cre-EBD to transfer into cell nucleus and bind to the loxPsym sites in the synthetic chromosomes, resulting an activated SCRaMbLE system.

**Fig. 2:**
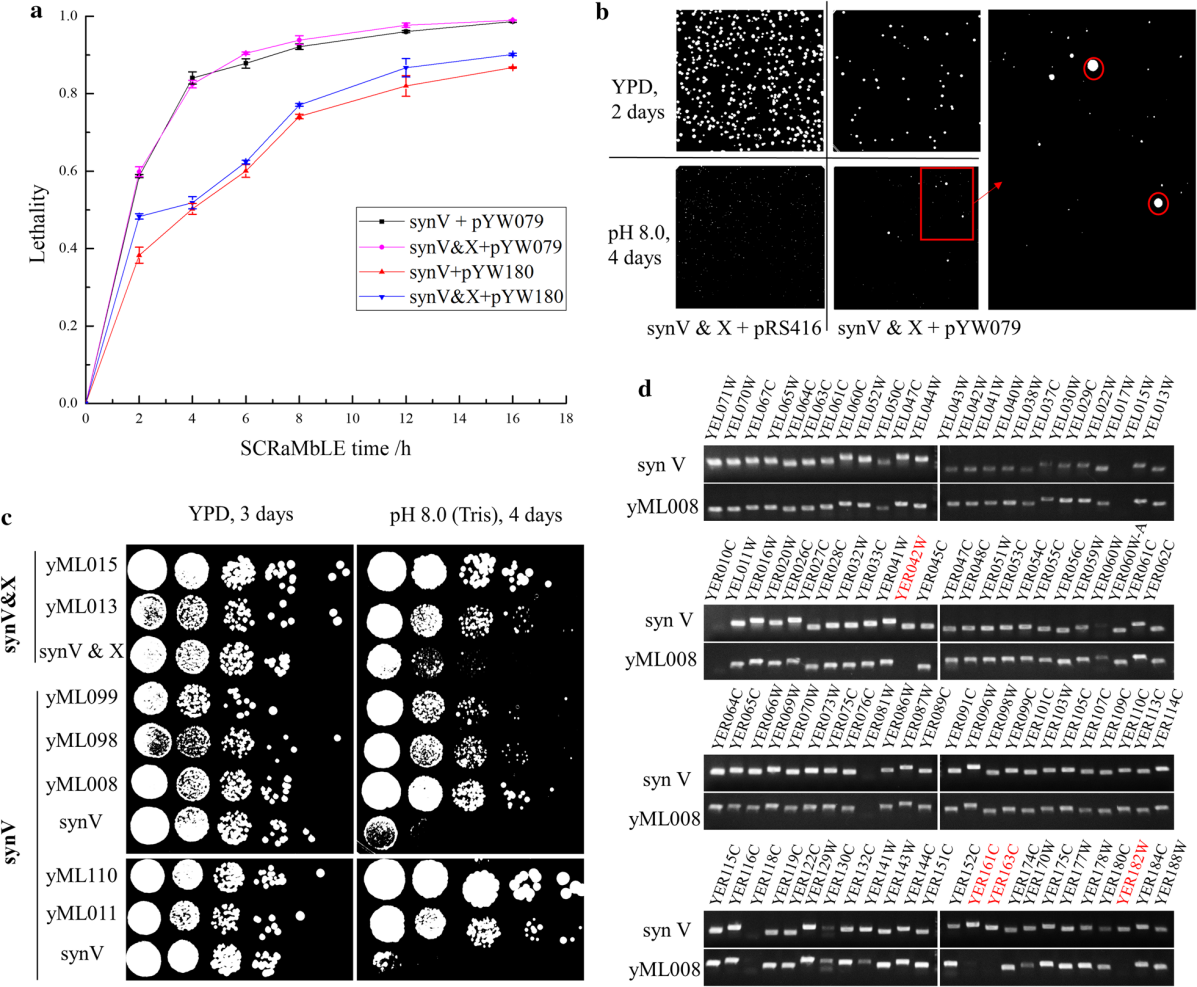
Characterization of SCRaMbLEd strains with increased alkali tolerance. **a** Characterization of lethality for SCRaMbLE system. Four strains (synV strain carrying pYW079, synV carrying pYW180, synV&X carrying pYW079 and synV&X carrying pYW079) were cultured in SC-Ura medium and exposed in β-estradiol for varied time (2 h, 4 h, 6 h, 8 h, 12 h, 16 h), and then diluted on YPD plates. SynV and synV&X strains containing empty vector pRS416 were used as control strains. **b** SCRaMbLEd synV&X strains on YPD medium and selective medium (YPD medium at pH 8.0). Strains on YPD medium were cultured 2 days and strains on selective medium were cultured 4 days in 30 °C before photographed. SynV&X strain containing empty vector pRS416 was used as a control strain. **c** Phenotype verification of SCRaMbLEd strains. SynV and synV&X strains were used as control strains. **d** PCRTag analysis. PCRTags of genes YER042W, YER161C, YER163C and YER182W were labeled in red, indicating deletions of these regions in strain yML008. None amplification of PCRTags of YEL017W, YER010C, YER081W, YER118C in both synV and yML008 strains was caused by nonspecific primers. SynV strain was used as a control strain. All PCRTag primers were listed in Additional file 1: Table S1

Although the loxPsym sites were inserted in the 3′ UTR of non-essential genes, essential genes might be deleted by Cre/loxPsym reaction across these genes, resulting the loss of viability for haploid synthetic yeasts. We characterized the lethality caused by SCRaMbLE firstly. As shown in Fig. 2a, lethality of strains carrying pYW079 was higher than strains carrying pYW180 due to the enhanced expression of Cre recombinase by a stronger promoter. And lethality of synV&X strains was slightly higher than synV strain. This may be caused by larger number of loxPsym sites within two synthetic chromosomes in synV&X strains. After 8 h’ SCRaMbLE, more than 70% lethality was detected in strains carried pYW180 and more than 90% lethality in strains carried pYW079. Then 8 h’ SCRaMbLE was considered as an appropriate intensity of genome evolution and chosen for the following SCRaMbLE experiments.

### SCRaMbLE generates evolved yeasts with increased alkali tolerance

YPD media with different pH were tested to select an appropriate alkaline condition for screening of SCRaMbLEd strains. pH 8.0 was chosen as the selective condition as showing enough alkaline pressure while keeping a reasonable culture time. Several bigger colonies from the SCRaMbLEd pool were detected though visual inspection on the selective plates after cultured at 30 °C for 4 days, while very few big colonies were grown on the selective plates from unSCRaMbLE pool (Fig. 2b). To avoid the possible leaky expression of Cre recombinase on candidate strains, the Cre-EBD plasmids were lost via serially culturing in YPD liquid medium. A total of seven strains (yML008, yML011, yML013, yML015, yML098, yML099 and yML110) generated through multiple independent SCRaMbLE experiments were verified with increased tolerance to alkali on YPD medium at pH 8.0 (Fig. 2c). Interestingly, SCRaMbLEd strains showed varied growth fitness under various stressful conditions (Additional file 1: Figure S1). Although all showed improved alkali tolerance, these strains were displayed with varied growth rate on the selective medium at pH 8.0 (i.e., yML008 grew faster than yML098 and yML099). For strain yML098, decreased growth fitness was detected on YPD medium at 30 °C, YP medium with 20 g L^−1^ xylose, and YPD medium with 1.5 M sorbitol. Meanwhile, some of SCRaMbLEd strains were displayed with increased growth fitness (Additional file 1: Figure S1, i.e., yML008 grown better than initial strain synV at 39 °C). These might be caused by diverse structural variations among the SCRaMbLEd strains.

The SCRaMbLEd genomes were preliminarily investigated by PCRTag method. The synthetic yeast chromosome V was divided into 177 segments by 176 loxPsym sites. 96 of distributed PCRTags at different loci were chosen to cover as many segments as possible (Additional file 1: Table S1). All PCRTags in segments encoding essential genes were deprecated considering low probability of deletion of these regions in haploid strains. A total of seven SCRaMbLEd strains with enhanced alkali tolerance were analyzed using the 96 PCRTags in the synthetic yeast chromosome V (Fig. 2d, Additional file 1: Figure S2). At least one PCRTag missing was found in yML008, yML013, yML015, yML098 and yML099 when compared to initial strain synV, indicating deletion events induced by SCRaMbLE. As shown in Fig. 2d, PCRTag analysis of strain yML008 indicated deletions of four genes including YER042W (*MXR1*), YER161C (*SPT2*), YER163C (*GCG1*), and YER182W (*FMP10*). However, other structural variations such as inversion, transposition, duplication and more complex genome rearrangements may not be detected by PCRTag assay directly.

### Complex structural variations generated by SCRaMbLE

**Fig. 3:**
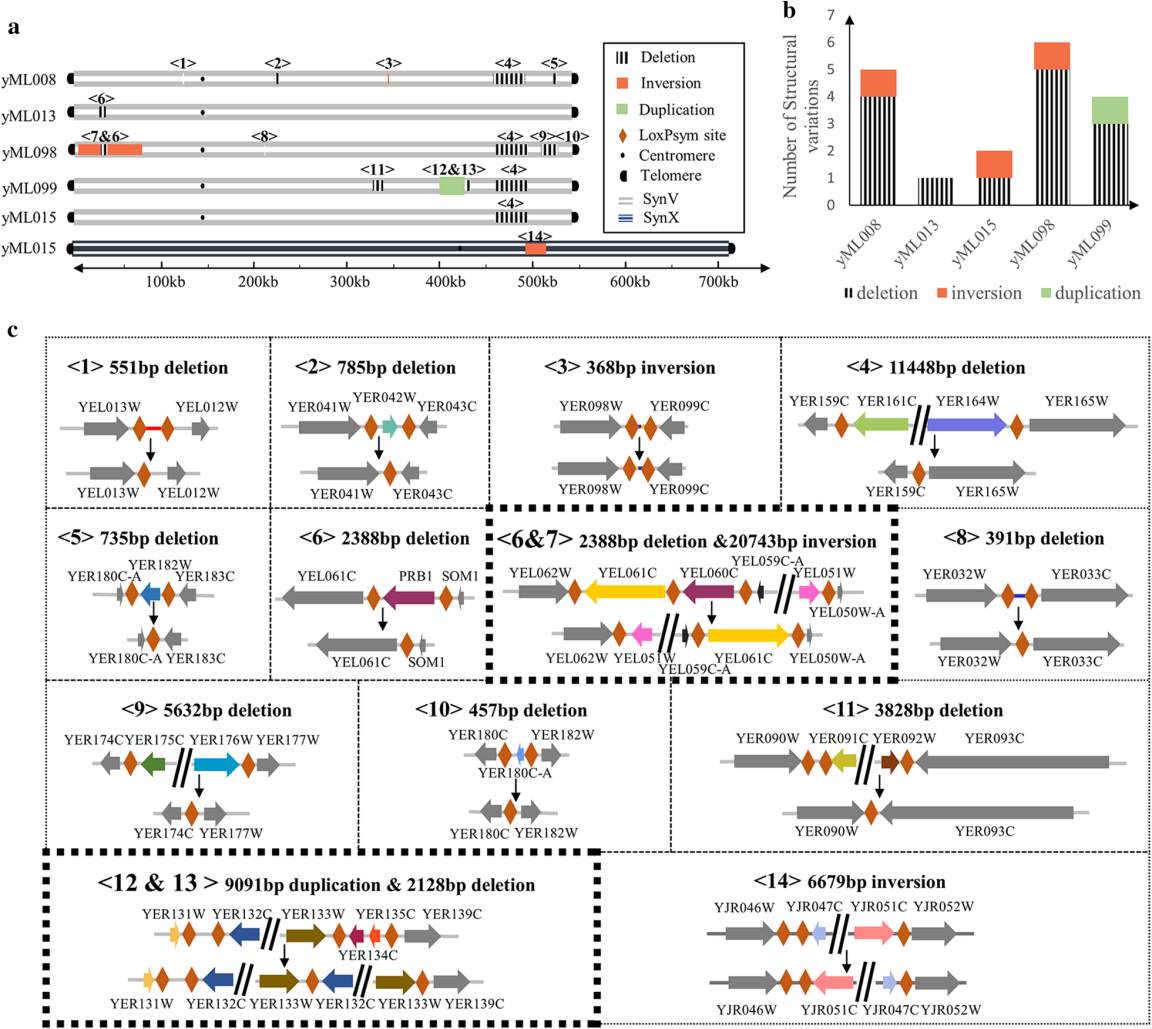
Analysis of structural variations in SCRaMbLEd strains. **a** Structural variations in five of the SCRaMbLEd genomes detected by whole genome sequencing. **b** Statistical analysis of structural variations in SCRaMbLEd genomes. **c** Detailed information of 14 varied rearrangement events. Two complex structural variations in section <6 and 7> and <12 and 13> were indicated with thick borders

All the seven SCRaMbLEd strains were further analyzed using whole genome sequencing to determine genomic variations driven by SCRaMbLE. Different types of structural variations including deletion, inversion, and duplication were detected in the SCRaMbLEd strains (Fig. 3a). Coverage maps of sequenced strains showed absence of reads in corresponding regions (Additional file 1: Figures S3–S9). There were at least four different rearranged events in strains yML008, yML098, and yML099 respectively. A maximum of six of rearranged events was generated in strain yML098 including five deletions and one inversion. Strains yML013 and yML015 generated from initial strain synV&X were detected with one or two structural variations. Statistical analysis of structural variations in SCRaMbLEd strains were shown in Fig. 3b. We speculated that strain synV&X providing more recombinational sites may result higher frequency of lethality caused by deletion of essential genes or synthetic lethal genetic interactions especially under the pressure of high pH.

We focused on novel junctions in the SCRaMbLEd genome based on data of sequence alignments. The sequencing result of strain yML098 revealed a complex structural variation which possessed an inversion of the region between YEL061C (*CIN8*) and YEL051W (*VMA8*) within which YEL060C (*PRB1*) was deleted simultaneously (Fig. 3a, c, region <6 and 7>). The total length of fragments deleted in strain yML098 reaches up to 19,965 base pairs, which is about 4% of the total length of synthetic yeast chromosome V. Another complex structural variation was also observed in the SCRaMbLEd genome of strain yML099 which contained a duplication region of 9091 base pairs with a deletion aside (Fig. 3a, c, region <12 and 13>). Besides, five of deletions (Fig. 3a, c, region <2>, <4>, <6>, <11> and <13>) were observed to be adjacent to an essential gene, indicating that essential genes may limit complex rearrangements across large fragments.

### Deletion of YE161C (*SPT2*) increases the alkali tolerance

**Fig. 4:**
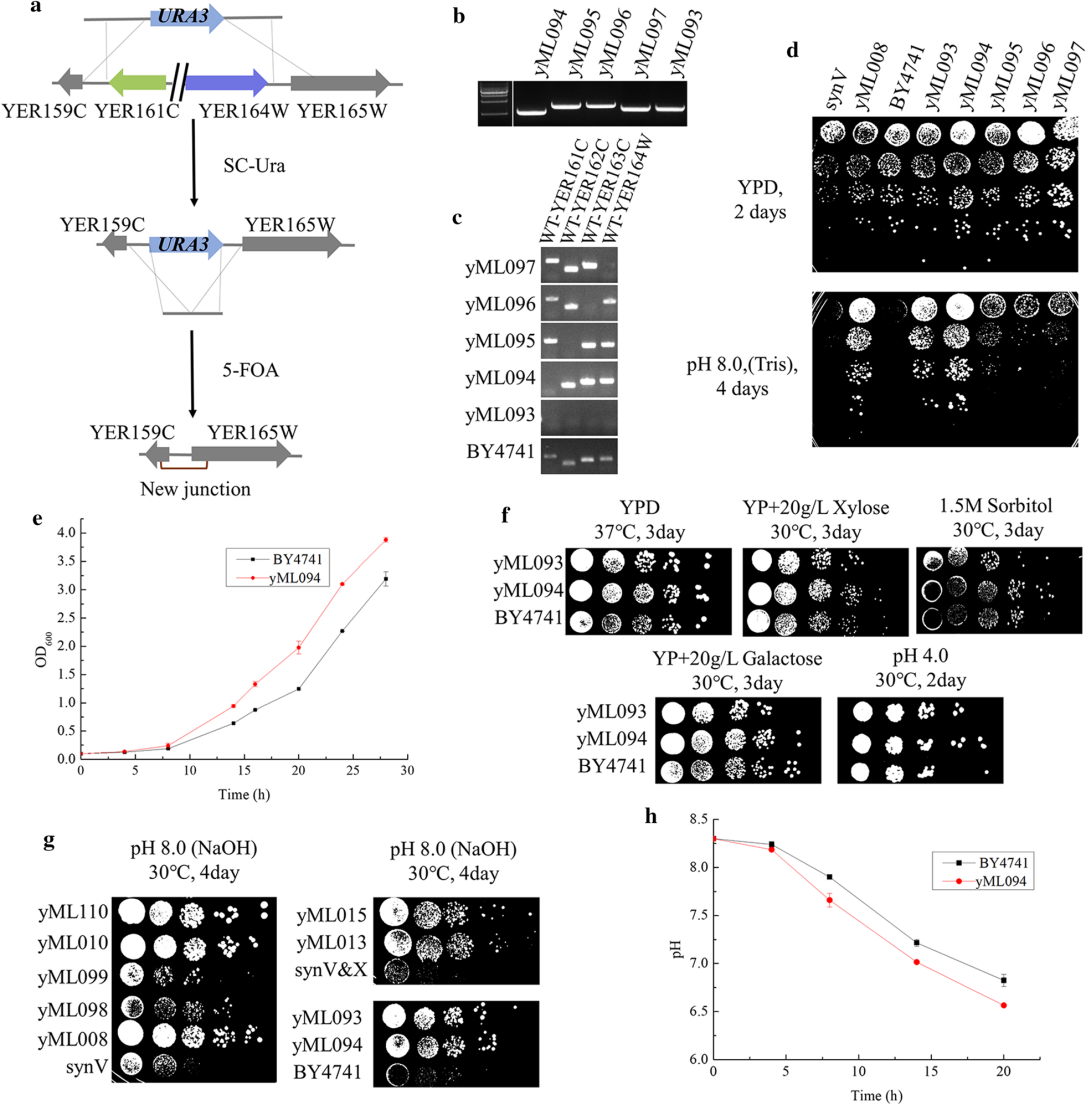
Deletion of YER161C (*SPT2*) increases the alkali tolerance in yeast. **a** Construction of seamless knockout yeast strains. With two rounds of genome integration, transformants were selected on SC-Ura and 5-FOA medium, respectively. **b** Verification of targeted deletions by junction PCR. All junction primers were listed in Additional file 1: Table S1. **c** Verification of individual gene deletions. All primers of individual genes were listed in Additional file 1: Table S1. **d** Characterization of the knockout yeast strains on YPD medium at pH 8.0. Strains on selective medium were cultured 4 days in 30 °C before photographed. **e** Growth curves of yML094 and BY4741 at pH 8.3. **f** Characterization of *SPT2* knockout strains under various stressful conditions. **g** Growth fitness of SCRaMbLEd strains and *SPT2* knockout strains at pH 8.0 using NaOH as alkali source. **h** pH changes of yML094 and BY4741 in liquid YPD medium at pH 8.3

One of the most obvious features among all structural variations observed in the SCRaMbLEd genome is the deletion of a region from YER161C to YER164W. This was commonly found in strains yML008, yML015, yML098 and yML099. The deleted region involved four genes with 11,448 base pairs in length. Therefore, we speculate that this region might be responsible for the enhanced phenotype of SCRaMbLEd strains under alkaline stress. To test the hypothesis above, we knocked out the relevant regions in wild type strain BY4741 which was the initial strain for both synthetic strains synV and synV&X. We constructed the knockout strains using the strategy shown in Fig. 4a. Strain yML093 with YER161C–YER164W deleted was verified by PCR (Fig. 4b, c). Compared to initial strain BY4741, yML093 had an enhanced phenotype at pH 8.0 which was similar to the SCRaMbLEd strains (Fig. 4d). We concluded that the deletion of the region YER161C–YER164W increased alkali tolerance of yeast strain BY4741 and was responsible for the enhanced phenotype of SCRaMbLEd strains yML008, yML015, yML098 and yML099 under alkaline pH. The varied degree of alkali tolerance among these SCRaMbLEd strains may be caused by other rearranged events in the SCRaMbLEd genomes. For instance, there were another four deletion regions in strain yML098. Among them, *ECM32* encodes a DNA helicase involving in modulating translation termination, and the deletion of *ECM32* results a decreased competitive fitness [36, 37].

To further narrow down the region responsible for the enhanced phenotype under alkali stress, we knocked out all four genes YER161C (*SPT2*), YER162C (*RAD4*), YER163C (*GCG1*) and YER164W (*CHD1*) within this region individually in strain BY4741. All four knockout strains were characterized on YPD medium at pH 8.0. Among them, strain yML094 with YER161C (*SPT2*) deleted showed a similar phenotype with the YER161C–YER164W deleted strain yML093, while the deletion of other 3 genes had no obvious influence on growth fitness of these strains under alkaline stress (Fig. 4d). Moreover, we quantified the tolerance to alkali condition by measuring growth curves of strains on YPD liquid medium at pH 8.3. Strain yML094 grows faster than BY4741 under alkali environment (Fig. 4e). These results reveal that YER161C (*SPT2*) might be a target gene responsible for increased alkali tolerance.

To further explore the mechanism of alkali tolerance caused by deletion of YER161C (*SPT2*), we tested growth fitness of the *SPT2* knockout strains on various stressful conditions. As shown in Fig. 4f, the *SPT2* deleted strains were displayed with similar growth fitness as control strain BY4741 under stressful conditions tested. It suggests that the mechanism of alkali tolerance caused by the deletion of *SPT2* is not suitable for other types of tolerance. The growth fitness of SCRaMbLEd strains and *SPT2* knockout strains were further evaluated at pH 8.0 using NaOH as alkali source (Fig. 4g). And the result is consistent with pervious data using Tris as alkali source (Figs. 2g and 4d). It suggests that the deletion of *SPT2* could enhance alkali tolerance specifically and not due to tolerance to Tris as a compound. We also tested the pH changes of cultured strains during the growth process with initial pH at 8.3 (Fig. 4h). The data indicates that *SPT2* knockout strains could accelerate the neutralization of the medium when compared with control strain BY4741. On the basis of previous studies, Spt2p is a DNA binding protein with HMG-like domains, and is required for RNA polyadenylation and involved in negative regulation of transcription [38–41]. Another study had found that the *SER3* mRNA level could be dramatically increased due to the deletion of *SPT2* [39]. Deletion of *SPT2* may change content of certain metabolites in cells or in the medium which may improve the growth fitness of yeast cell under alkaline environment. Although the exact underlying mechanism is not clear yet, deletion of YER161C (*SPT2*) enhanced the alkali tolerance indeed.

For other evolved strains without deletion of *SPT2* (yML011, yML013 and yML110), results of genome sequencing revealed very few structural variations (Additional file 1: Figures S4, S5, S9). However, a few single nucleotide polymorphisms (SNPs) were detected in the evolved genomes (i.e. 78 for yML110, 141 for yML011, 71 for yML013). These point mutations might be responsible for increased alkali tolerance of the evolved strains.

Our understanding of complex phenotypes (i.e., growth, tolerance) is limit mainly due to the absence of efficient multiplex engineering tools in genome scale [31, 42]. The strategy of using SCRaMbLE system to generate diverse and combinational rearrangements in the synthetic yeast genome provides a powerful platform to dissect phenotype-genotype relationship.

## Conclusions

The SCRaMbLE system enables generation of a yeast structural variation library in a short time with the expression of Cre recombinase. In this study, alkali tolerant strains were obtained via one round of SCRaMbLE in synthetic yeast strains synV and synV&X. Various combinations of deletion, inversion and duplication were detected in the SCRaMbLEd genome of strains with alkali tolerance. Through comparative analysis of structural variations in the SCRaMbLEd genome, a deletion region with 11,448 base pairs in length was mapped to be responsible for the enhanced phenotype. Furthermore, we validated the deletion of *SPT2* gene within the mapped region could improve alkali tolerance in *S. cerevisiae*. This study extends our knowledge of alkali tolerance in yeast and provides a standard workflow for the application of SCRaMbLE system to generate various phenotypes that may be interesting for industry and extend understanding of phenotype-genotype relationship.

## Methods

### Strains, plasmids and media

Plasmids and yeast strains used in this study were listed in Table 1. pRS416-pSCW11-Cre-EBD and pRS416-pCLB2-Cre-EBD are available from Addgene. Synthetic strains synV (*MATa*, *his3Δ1*, *leu2Δ0*, *met15Δ0*, *ura3Δ0*, *LYS2*) and synV&X (*MATa*, *his3Δ1*, *leu2Δ0*, *met15Δ0*, *ura3Δ0*, *HO::tR(ccu)J*, *lys::NAT*) were subjected to SCRaMbLE experiments. Wild type strain BY4741 (*MATa*, *his3Δ1*, *leu2Δ0*, *met15Δ0*, *ura3Δ*0) was used to test targets of alkali tolerance mapped by SCRaMbLE in synthetic yeast. Yeast strains were grown in YPD medium containing 20 g L^−1^ glucose, 20 g L^−1^ peptone and 10 g L^−1^ yeast extract. SC-Ura (synthetic media lacking uracil) medium with 1 μM β-estradiol (Sigma-Aldrich) was used to induce SCRaMbLE. Selective medium for alkali tolerance was YPD medium at pH 8.0. 1 M Tris buffer or 2 M NaOH was used to adjust the pH, and glucose was added after sterilization at high temperature. SC medium contained 1 g L^−1^ 5-FOA (Sigma-Aldrich) was used to screen strains without *URA3* marker. All yeast solid media were added with 20 g L^−1^ agar. *Escherichia coli* DH5α purchased from BEIJING Biomed Co., Ltd were used for plasmids transformation. *Escherichia coli* were cultivated at 37 °C in LB medium with 10 g L^−1^ tryptone, 5 g L^−1^ yeast extract, and 10 g L^−1^ NaCl. 100 μg mL^−1^ ampicillin or kanamycin were added for selection. LB solid medium were added with 15 g L^−1^ agar.

**Table 1:** Strains and plasmids used in this study

Strain and plasmid	Description
yXZX846	Haploid strain synV
yYW169	Haploid strain SynV&X
yML008	SCRaMbLEd strain with enhanced alkali tolerance from yXZX846
yML011	SCRaMbLEd strain with enhanced alkali tolerance from yXZX846
yML013	SCRaMbLEd strain with enhanced alkali tolerance from yYW169
yML015	SCRaMbLEd strain with enhanced alkali tolerance from yYW169
yML098	SCRaMbLEd strain with enhanced alkali tolerance from yXZX846
yML099	SCRaMbLEd strain with enhanced alkali tolerance from yXZX846
yML110	SCRaMbLEd strain with enhanced alkali tolerance from yXZX846
yML092	yYW0169 with YER161C-164W deleted
yML093	BY4741 with YER161C-164W deleted
yML094	BY4741 with YER161C deleted
yML095	BY4741 with YER162C deleted
yML096	BY4741 with YER163C deleted
yML097	BY4741 with YER164W deleted
pRS416	*CEN*/*ARS* with *URA3* marker
pYW079	pRS416-SCW11-Cre-EBD
pYW180	pRS416-CLB2-Cre-EBD

### pH tolerance test of the synV and synV&X strains

To identify a suitable pH selective condition that synthetic strains synV and synV&X could survive but have a limited growth speed, we used the serial dilution assay. According to a previous study [30], YPD, YPD at pH 7.4, YPD at pH 7.6, YPD at pH 7.8, YPD at pH 8.0 and YPD at pH 8.2 were used for the test.

### Yeast transformation

LiAc/SS carrier method was used for yeast transformation. Strains grown overnight in YPD were diluted to OD_600_ of 0.1 in fresh YPD and cultured to exponential phase (5–8 h) at 30 °C. Cells were washed once with ddH_2_O, resuspended in 0.1 M LiAc and put on ice until needed. Yeast transformation system contained 620 μL 50% polyethylene glycol (PEG-3350), 40 μL salmon sperm DNA (100 mg mL^−1^), 90 μL 1 M LiAc, and 150 μL mixture of plasmids or fragments and cells. The system needed to be incubated at 30 °C for 30 min. 90 μL DMSO was added followed by heat-shocked at 42 °C for 18 min. Centrifuged and resuspended cells with 5 mM CaCl_2_, plated on selective medium. For plasmid, 100 ng was enough for transformation. Both pYW079 (pRS416-SCW11-Cre-EBD) and pYW180 (pRS416-pCLB2-Cre-EBD) plasmids were transformed to haploid strains synV and synV&X for SCRaMbLE. After culturing for 72 h at 30 °C, correct colonies could be selected on SC-Ura plates. As for fragments integrated to genome, at least 300 ng was required.

### SCRaMbLE

First, single colony was cultured in 5 mL SC-Ura at 30 °C overnight. Then cultures were diluted to OD_600_ of 0.1 and inoculated to 5 mL fresh SC-Ura. 1 μM β-estradiol was added to induce SCRaMbLE. After incubated at 30 °C for 8 h, cells were washed twice with ddH_2_O to wash out β-estradiol. Finally, cells were diluted to spot onto both YPD plates and selective plates. The number of colonies on YPD was counted to figure out the lethality. Selective plates were incubated at 30 °C for 4–5 days.

### Screening and verification of alkali tolerance strains

For preliminary screening, big colonies were selected on the selective media at pH 8.0. Then the candidate colonies were serially cultured in liquid YPD to lose the Cre-EBD plasmid. After that, candidate strains were phenotypically verified on YPD media at pH 8.0 using tenfold serial dilution assay.

### Extraction of the yeast genomic DNA

Strains were cultured overnight to saturation. Centrifuged at 12,000 rpm to harvest cells. 200 μL STES buffer (500 mM L^−1^ NaCl, 200 mM L^−1^ Tris–HCl, 100 mM L^−1^ EDTA, 1% SDS), 200 μL silica sand and 200 μL phenol/chloroform/isoamyl alcohol (25:24:1) were added to cells. Disrupted cells by vortex mixer for 10 min. Then added 1 mL ethanol to the supernatant, mixed and centrifuged at 4 °C for 10 min. Precipitate was washed with 75% ethanol and dried at 42 °C. 100 μL ddH_2_O was added to dissolve the yeast genome DNA. Stored the genome DNA at − 20 °C.

### PCRTagging analysis

Fifteen microlitre PCR reaction system contained 7.5 μL 2X rapid Taq master mix (Vazyme), 0.4 μL forward primer (10 μM), 0.4 μL reverse primer (10 μM), 1 μL genome DNA, and 5.7 μL ddH_2_O. The procedure: 95 °C/1 min, 30 cycles of (95 °C/20 s, 53 °C/20 s, 72 °C/15 s), and 72 °C/5 min. Agarose gel electrophoresis was used for PCRTag analysis. Primers involved in this study were listed Additional file 1: Table S1.

### Whole genome sequencing

Cells were harvested at exponential phase and sent to BGI (the Beijing Genomics Institute) or BIOMARKER TECHNOLOGIES for whole genome sequencing. Libraries were prepared and performed on Illumina (HiSeq X-Ten). The original data obtained were filtered. SOAPaligner were used for average depth analysis. Through comparing with reference sequences, SNP, InDel and SV could be detected. The SVs including insertion, deletion, inversion, intra-chromosomal translocation, and inter-chromosomal translocation were analyzed.

### Deletion of target genes

Genes were deleted by homologous recombination. The deletion of gene YER161C (*SPT2*) was used as an example to explain the strategy. Homologous arms of upstream and downstream of YER161C (*SPT2*) gene were amplified from the genome of BY4741. Selective marker *URA3* was amplified from pRS416. Then three parts were joined together by overlap PCR and 300 ng of gel purified DNA fragments were directly transformed into BY4741 on SC-Ura plates. DNA fragment with homologous arms of gene YER161C (*SPT2*) was used to replace the marker *URA3* by the same assay to avoid the effect of *URA3* gene on the phenotypes of alkali tolerance. Correct transformants could be selected on 5-FOA plates. Other genes (YER162C, YER163C, YER164W, YER161C–YER164W) were deleted in the same way.

### Growth curve assay

Single colony was cultured to saturation in 5 mL YPD medium at 30 °C. The cultures were inoculated into a 250 mL shake flask containing 50 mL of YPD medium at pH 8.3 (34 mM Tris L^−1^) with initial OD_600_ at 0.1, and cultured at 30 °C, 220 rpm. The OD value was measured at appropriate intervals. Growth curves were plotted using Origin software.

## Additional file

**Additional file 1: Figure S1.** Stress tolerance of SCRaMbLEd strains. SCRaMbLEd strains were tested under various stressful conditions (YPD medium at 30 °C, YPD medium at 37 °C, YPD medium at 39 °C, YP medium with 20 g/L Xylose, YPD medium with 1.5 M Sorbitol and YP medium with 20 g/L Galactose). The growth of SCRaMbLEd strains was evaluated based on serial dilution. Two independent experiments were performed. **Figure S2.** PCRTag analysis of SCRaMbLEd strains. PCRTag analysis indicated deletion of YEL060C in strain yML013, deletions of YER161C and YER163C in strain yML015, deletions of YEL060C, YER161C, YER163C and YER175C in strain yML077, deletions of YER091C, YER161C and YER163C in yML099. No PCRTags were deleted in yML011 and yML110. SynV strain was used as a control strain. All PCRTag primers were listed in Table S1. **Figure S3.** Sequencing depth of synthetic chromosome V in yML008. Deep sequencing coverage of yML008 revealed four deletions (an intergenic sequence between YEL013W and YEL012W, YER042W, YER161C-YER164W and YER182W). **Figure S4.** Sequencing depth of synthetic chromosome V in yML011. Deep sequencing coverage revealed no synthetic fragments deleted in yML011. **Figure S5.** Sequencing depth of synthetic chromosomes in yML013. **a** Deep sequencing coverage of synthetic chromosome V in yML013 revealed a deletion of YEL060C. **b** Deep sequencing coverage revealed no synthetic fragments deleted in synthetic chromosome X in yML013. **Figure S6.** Sequencing depth of synthetic chromosomes in yML015. **a** Deep sequencing coverage of synthetic chromosome V in yML015 revealed a deletion of YER161C-YER164W. **b** Deep sequencing coverage revealed no synthetic fragments deleted in synthetic chromosome X in yML015. **Figure S7.** Sequencing depth of synthetic chromosome V in yML098. Deep sequencing coverage of yML098 revealed five deletions (YEL060C, an intergenic sequence between YER032W and YEL033C, YER161C-YER164W, YER175C-YER176W, and YER180C-A). **Figure S8.** Sequencing depth of synthetic chromosome V in yML099. Deep sequencing coverage of yML099 revealed three deletions (YER091C-YER092W, YER134C-YER135C, YER161C-YER164W) and one duplication (YER132C-YER133W). **Figure S9.** Sequencing depth of synthetic chromosome V in yML110. Deep sequencing coverage revealed no synthetic fragments deleted in yML110. **Table S1.** Primers used in this study.

## Abbreviations

SCRaMbLE: synthetic chromosome recombination and modification by LoxP-mediated evolution
Sc 2.0 project: synthetic yeast genome project
3′UTR: 3′ untranslated region
EBD: estrogen-binding domain
