# Supplementary material for: SCRaMbLE generates evolved yeasts with increased alkali tolerance
Source: Microb Cell Fact. 2019 Mar 11;18:52. doi: 10.1186/s12934-019-1102-4 (PMC6410612; doi:10.1186/s12934-019-1102-4)
Supplement: Supplementary file 1 — Additional file 1: Figure S1. Stress tolerance of SCRaMbLEd strains. SCRaMbLEd strains were tested under various stressful conditions (YPD medium at 30 °C, YPD medium at 37 °C, YPD medium at 39 °C, YP medium with 20 g/L Xylose, YPD medium with 1.5 M Sorbitol and YP medium with 20 g/L Galactose). The growth of SCRaMbLEd strains was evaluated based on serial dilution. Two independent experiments were performed. Figure S2. PCRTag analysis of SCRaMbLEd strains. PCRTag analysis indicated deletion of YEL060C in strain yML013, deletions of YER161C and YER163C in strain yML015, deletions of YEL060C, YER161C, YER163C and YER175C in strain yML077, deletions of YER091C, YER161C and YER163C in yML099. No PCRTags were deleted in yML011 and yML110. SynV strain was used as a control strain. All PCRTag primers were listed in Table S1. Figure S3. Sequencing depth of synthetic chromosome V in yML008. Deep sequencing coverage of yML008 revealed four deletions (an intergenic sequence between YEL013W and YEL012W, YER042W, YER161C-YER164W and YER182W). Figure S4. Sequencing depth of synthetic chromosome V in yML011. Deep sequencing coverage revealed no synthetic fragments deleted in yML011. Figure S5. Sequencing depth of synthetic chromosomes in yML013. a Deep sequencing coverage of synthetic chromosome V in yML013 revealed a deletion of YEL060C. b Deep sequencing coverage revealed no synthetic fragments deleted in synthetic chromosome X in yML013. Figure S6. Sequencing depth of synthetic chromosomes in yML015. a Deep sequencing coverage of synthetic chromosome V in yML015 revealed a deletion of YER161C-YER164W. b Deep sequencing coverage revealed no synthetic fragments deleted in synthetic chromosome X in yML015. Figure S7. Sequencing depth of synthetic chromosome V in yML098. Deep sequencing coverage of yML098 revealed five deletions (YEL060C, an intergenic sequence between YER032W and YEL033C, YER161C-YER164W, YER175C-YER176W, and YER180C-A). Figure S8. Sequencing depth of synthetic c [file 12934_2019_1102_MOESM1_ESM.docx]

**Additional file**

**SCRaMbLE generates evolved yeasts with increased alkali tolerance**

Lu Ma^1,2^, Yunxiang Li^1,2^, Xinyu Chen^1,2^, Mingzhu Ding^1,2^, Yi Wu^1,2*^, Yingjin Yuan^1,2^

^1^Frontier Science Center for Synthetic Biology and Key Laboratory of Systems Bioengineering (Ministry of Education), School of Chemical Engineering and Technology, Tianjin University, Tianjin 300072, China

^2^Collaborative Innovation Center of Chemical Science and Engineering (Tianjin), Tianjin University, Tianjin 300072, China

* To whom all correspondence should be addressed:

Dr. Yi Wu

Email: yi.wu@tju.edu.cn

Email addresses for all authors: [malu@tju.edu.cn](mailto:malu@tju.edu.cn); [liyunxiangcool@163.com](mailto:liyunxiangcool@163.com); [ccchenxy@tju.edu.cn](mailto:ccchenxy@tju.edu.cn); [mzding@tju.edu.cn](mailto:mzding@tju.edu.cn); [yi.wu@tju.edu.cn](mailto:yi.wu@tju.edu.cn); [yjyuan@tju.edu.cn](mailto:yjyuan@tju.edu.cn).

**
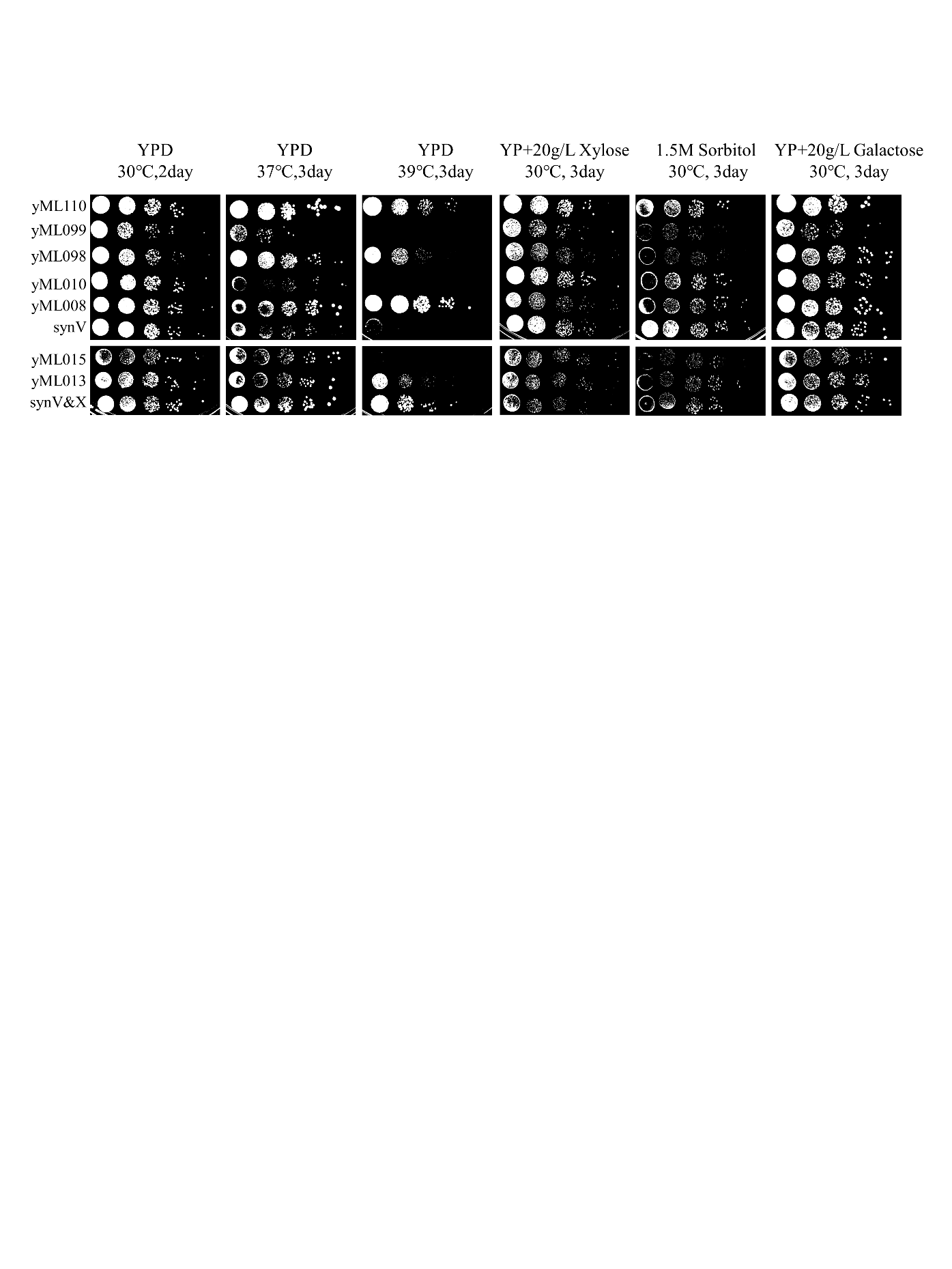
Figures**

**Fig. S1 Stress tolerance of SCRaMbLEd strains.** SCRaMbLEd strains were tested under various stressful conditions (YPD medium at 30℃, YPD medium at 37℃, YPD medium at 39℃, YP medium with 20g/L Xylose, YPD medium with 1.5M Sorbitol and YP medium with 20g/L Galactose). The growth of SCRaMbLEd strains was evaluated based on serial dilution. Two independent experiments were performed.

**
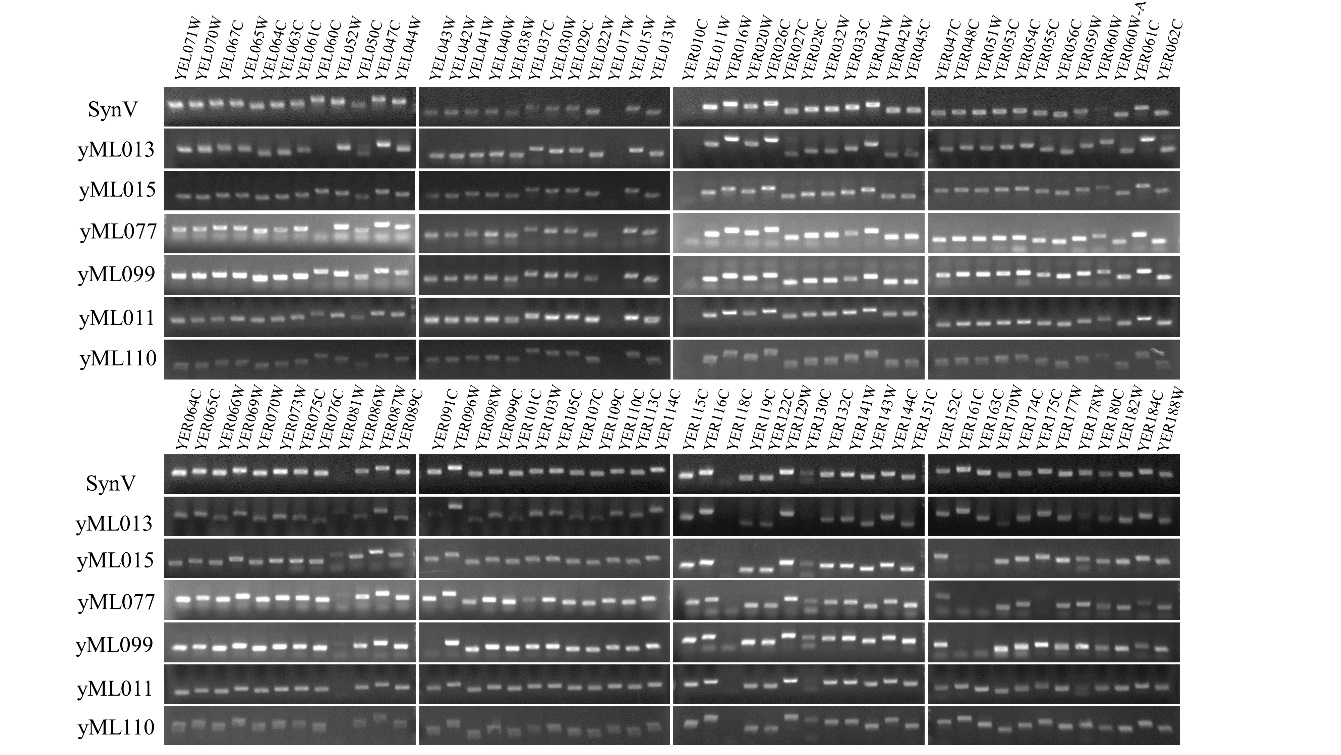
**

**Fig. S2 PCRTag analysis of SCRaMbLEd strains.** PCRTag analysis indicated deletion of YEL060C in strain yML013, deletions of YER161C and YER163C in strain yML015, deletions of YEL060C, YER161C, YER163C and YER175C in strain yML077, deletions of YER091C, YER161C and YER163C in yML099. No PCRTags were deleted in yML011 and yML110. SynV strain was used as a control strain. All PCRTag primers were listed in Table S1.

**
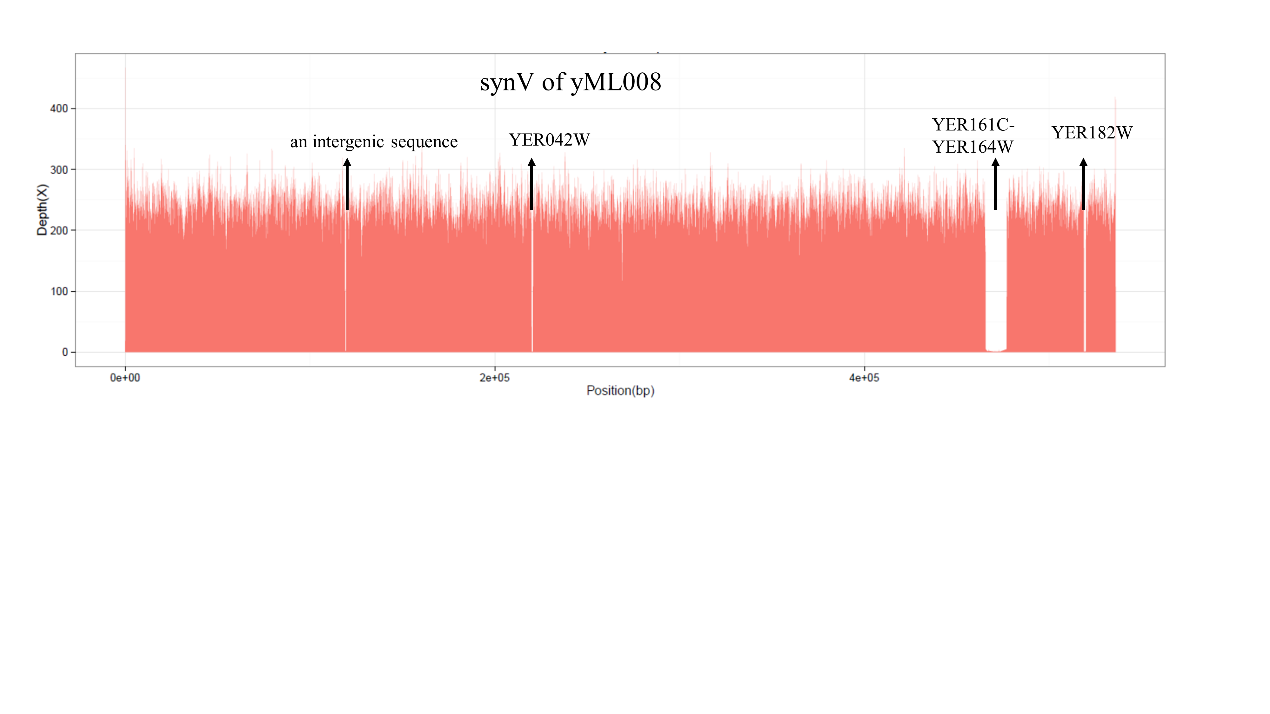
**

**Fig. S3 Sequencing depth of synthetic chromosome V in yML008.** Deep sequencing coverage of yML008 revealed four deletions (an intergenic sequence between YEL013W and YEL012W, YER042W, YER161C-YER164W and YER182W).


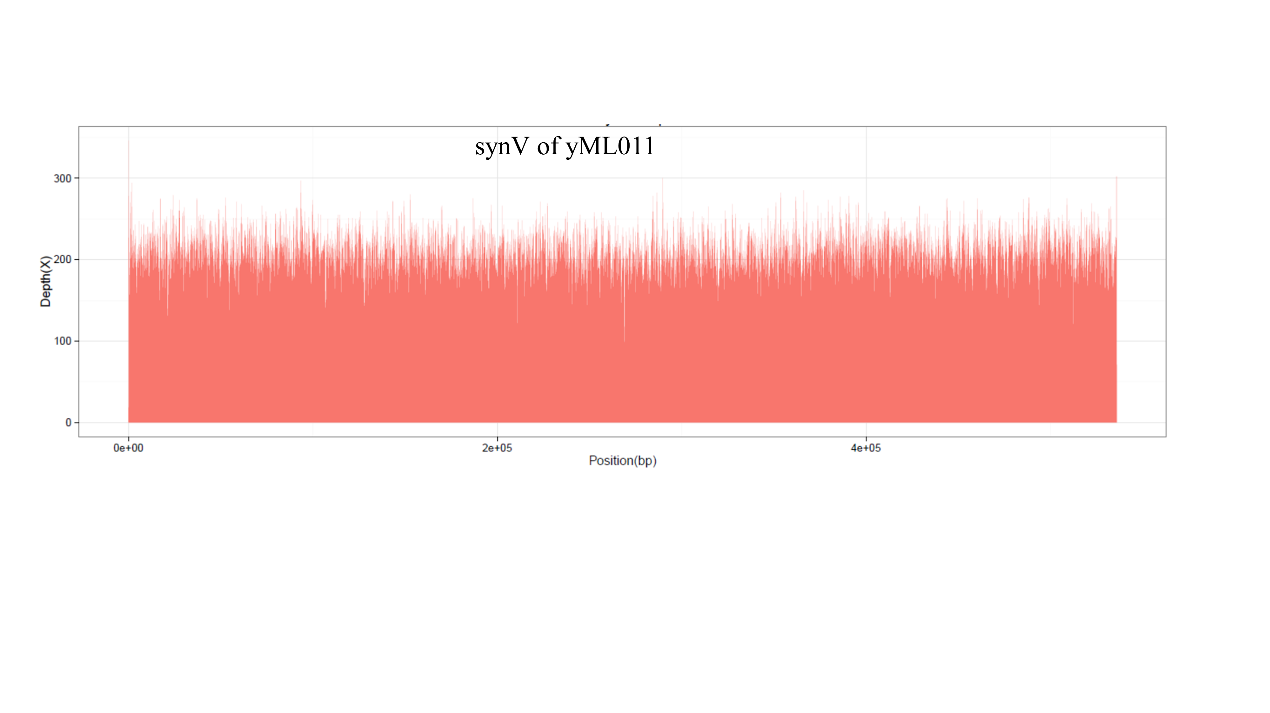


**Fig. S4 Sequencing depth of synthetic chromosome V in yML011.** Deep sequencing coverage revealed no synthetic fragments deleted in yML011.


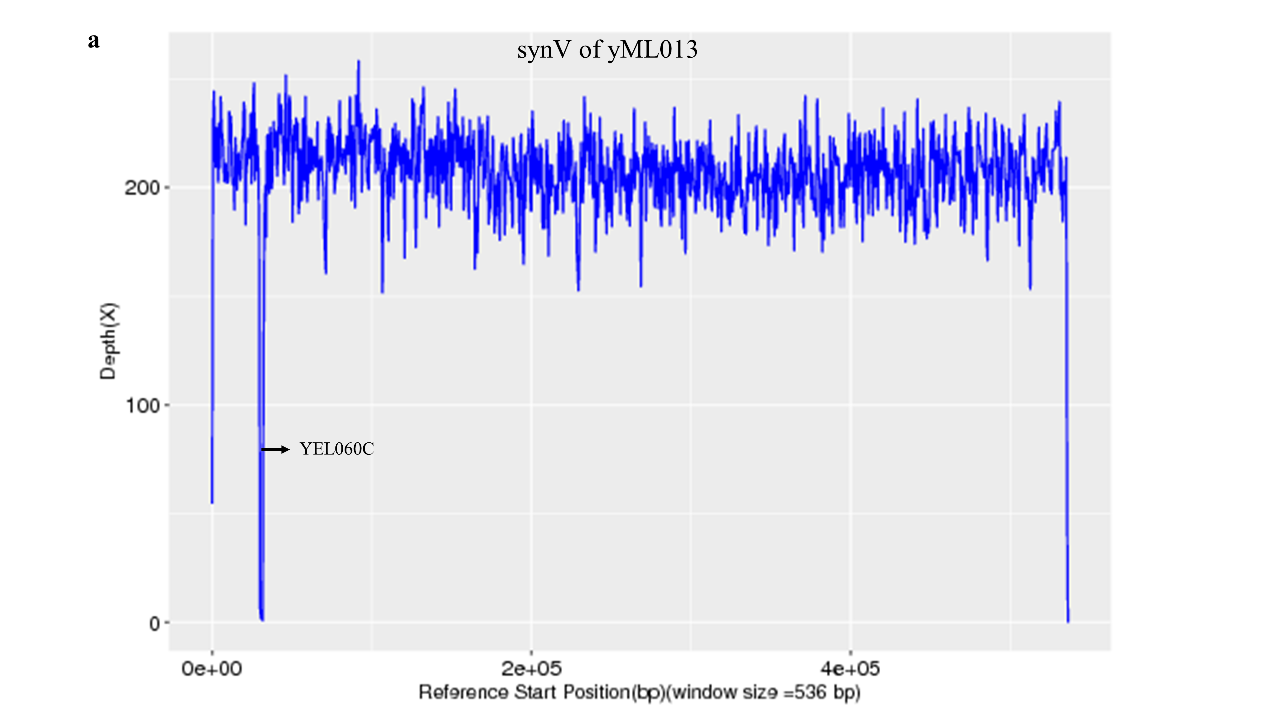

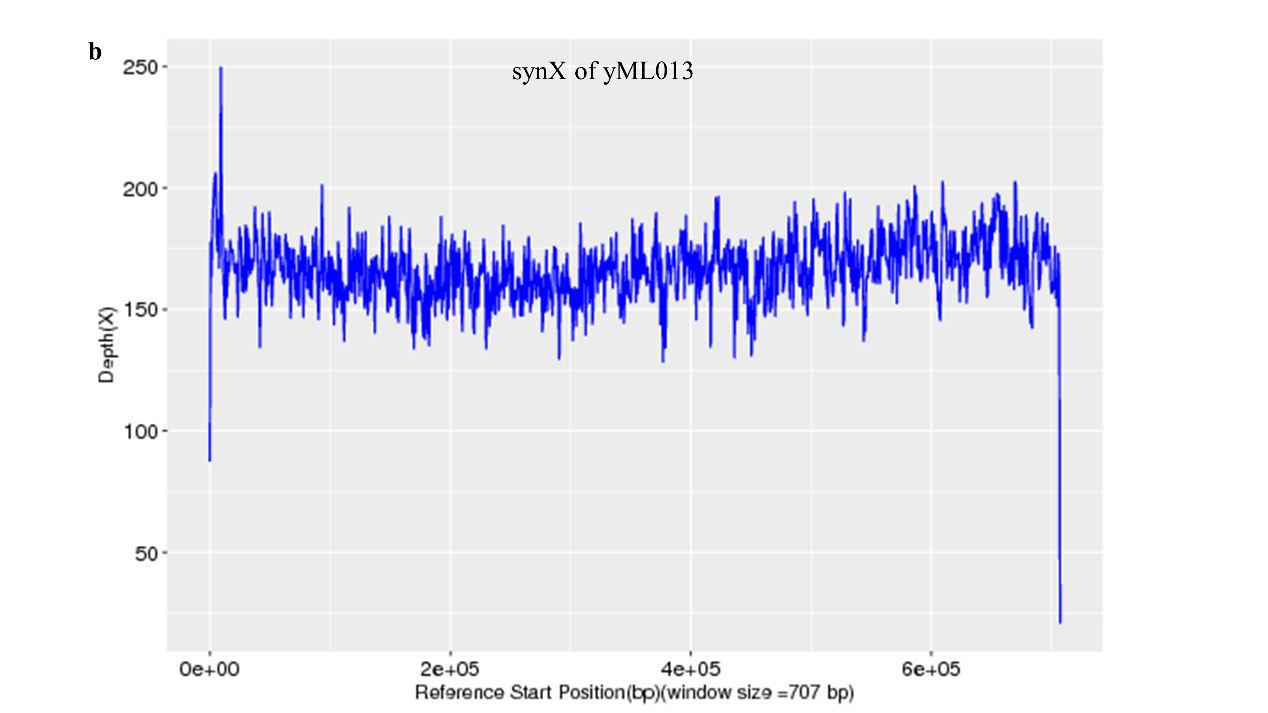


**Fig. S5** **Sequencing depth of synthetic chromosomes in yML013. a** Deep sequencing coverage of synthetic chromosome V in yML013 revealed a deletion of YEL060C. **b** Deep sequencing coverage revealed no synthetic fragments deleted in synthetic chromosome X in yML013.


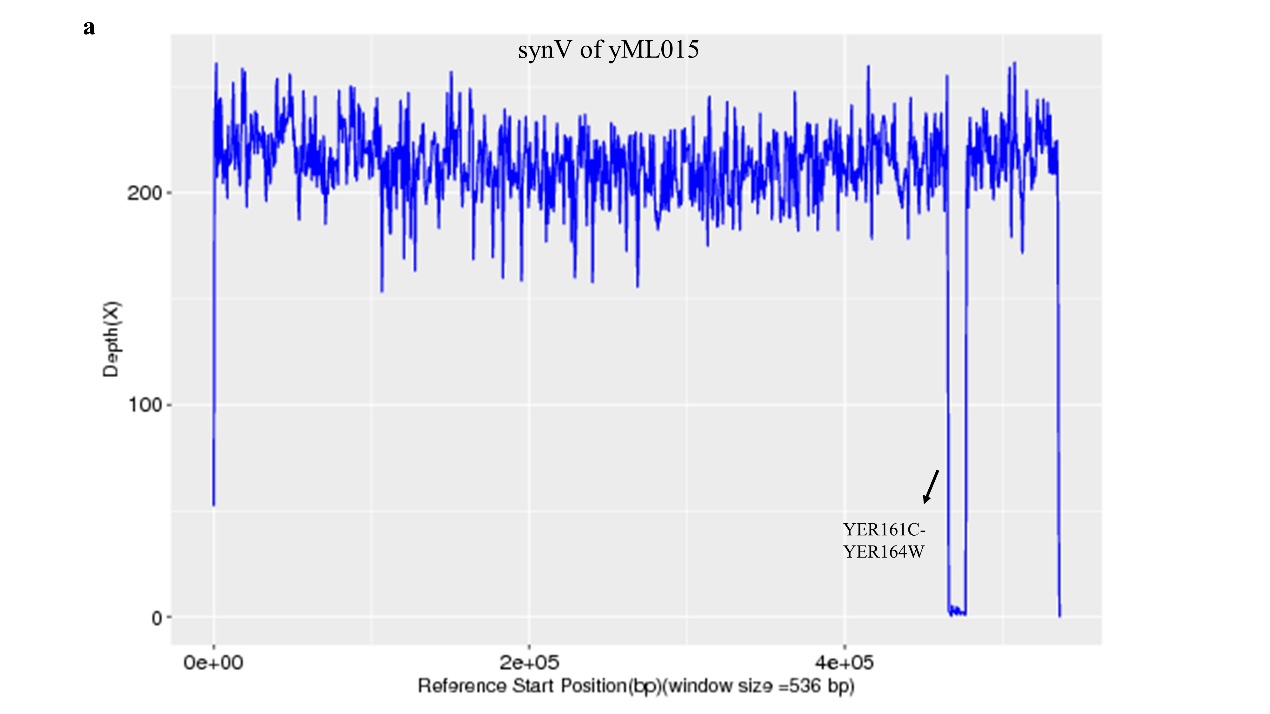

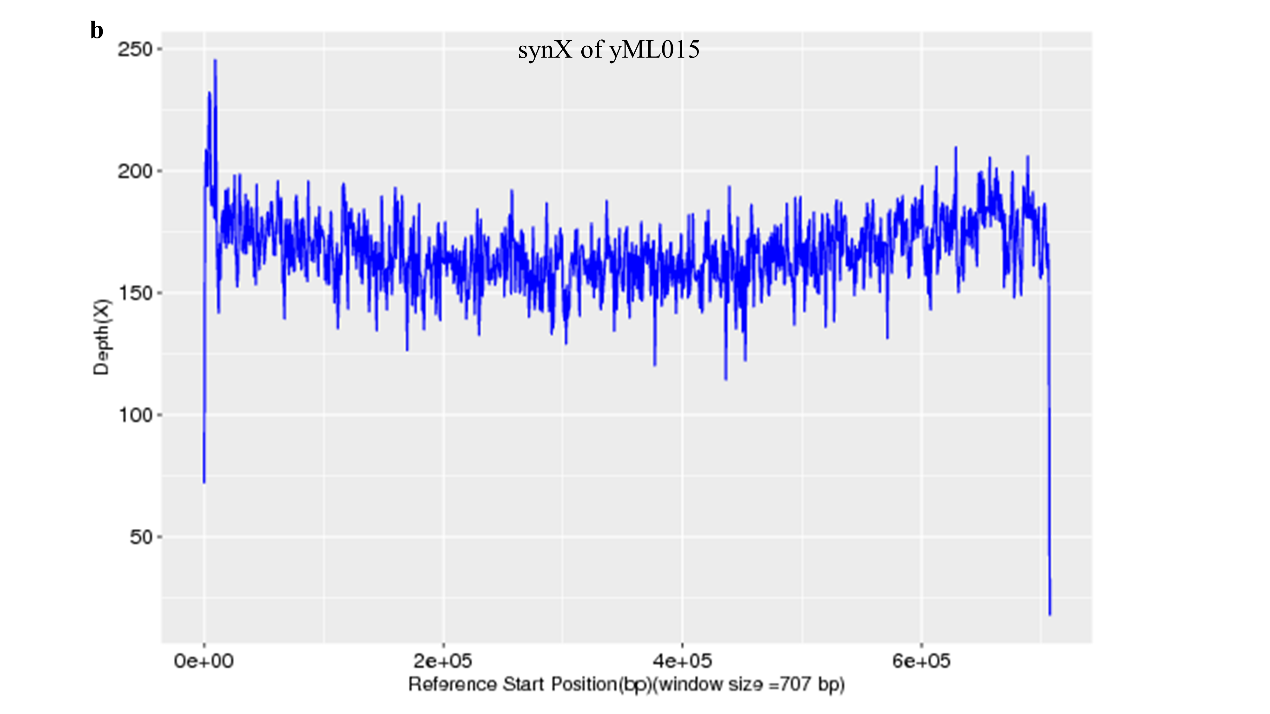


**Fig. S6** **Sequencing depth of synthetic chromosomes in yML015. a** Deep sequencing coverage of synthetic chromosome V in yML015 revealed a deletion of YER161C-YER164W. **b** Deep sequencing coverage revealed no synthetic fragments deleted in synthetic chromosome X in yML015.

**
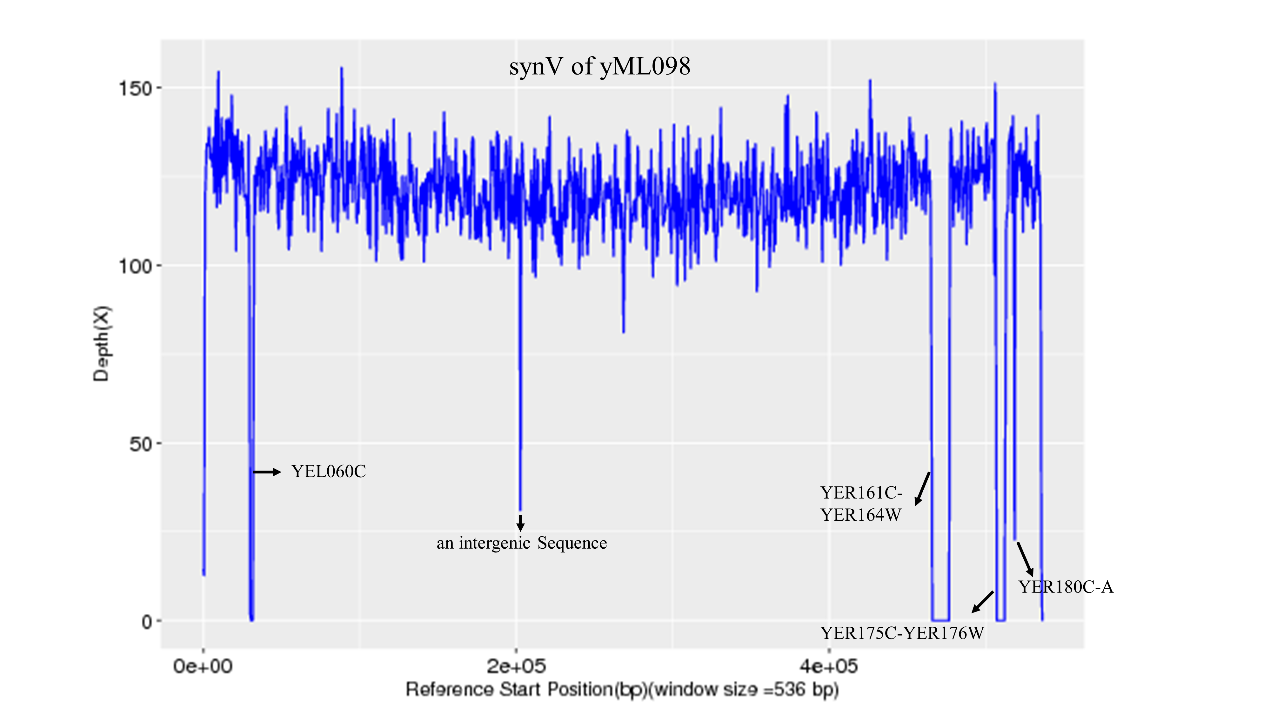
**

**Fig. S7** **Sequencing depth of synthetic chromosome V in yML098.** Deep sequencing coverage of yML098 revealed five deletions (YEL060C, an intergenic sequence between YER032W and YEL033C, YER161C-YER164W, YER175C-YER176W, and YER180C-A).

**
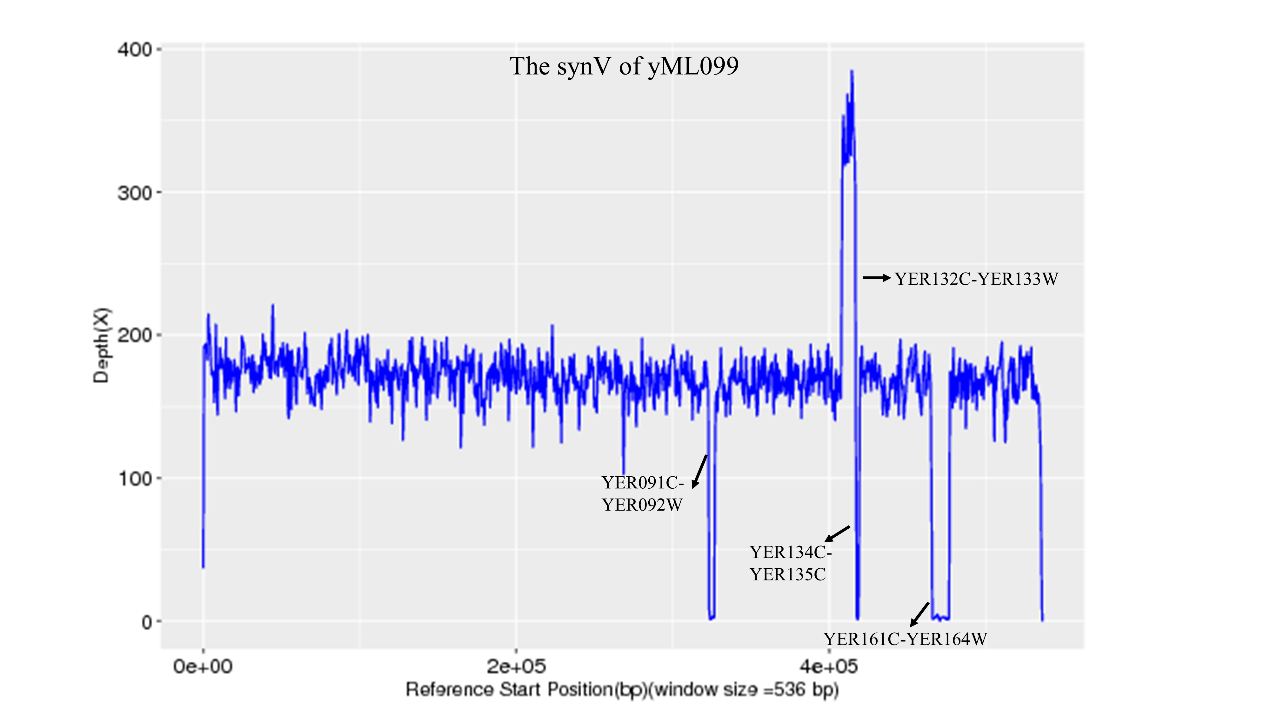
**

**Fig. S8** **Sequencing depth of synthetic chromosome V in yML099.** Deep sequencing coverage of yML099 revealed three deletions (YER091C-YER092W, YER134C-YER135C, YER161C-YER164W) and one duplication (YER132C-YER133W).

**
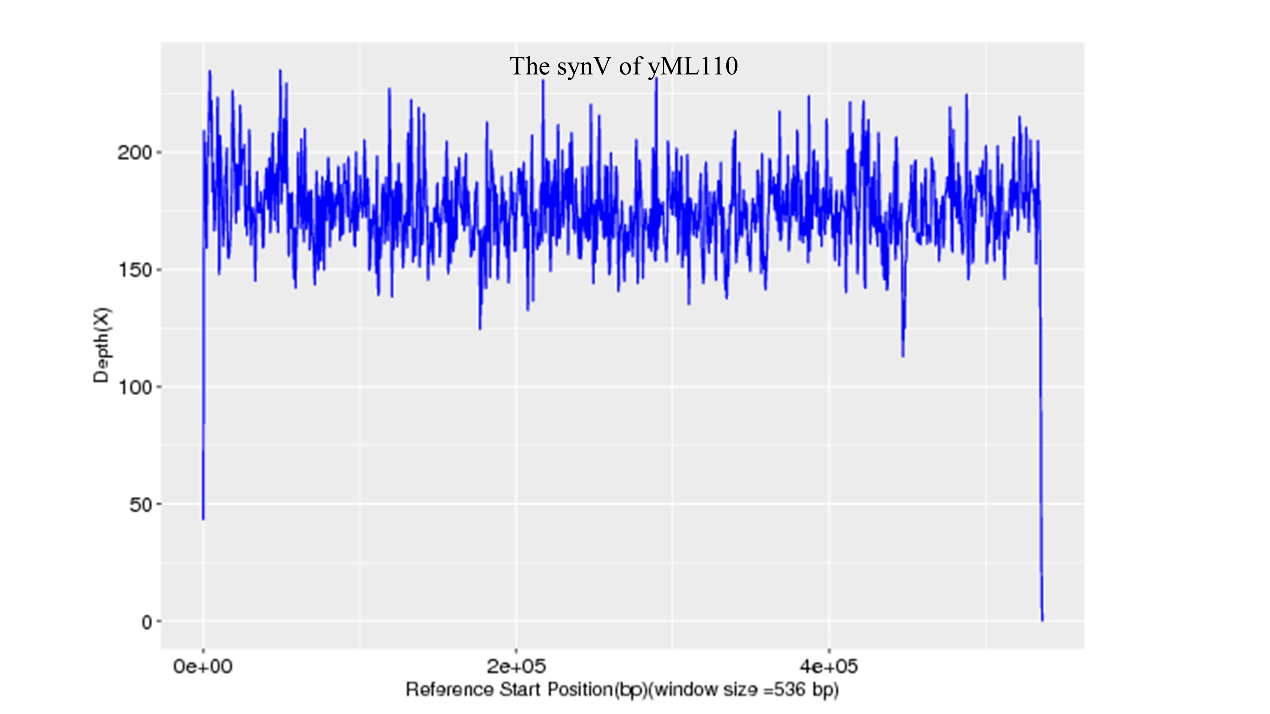
**

**Fig. S9** **Sequencing depth of synthetic chromosome V in yML110.** Deep sequencing coverage revealed no synthetic fragments deleted in yML110.

**Table**

**Table S1** Primers used in this study

| Name | Sequences | Description |
| --- | --- | --- |
| YEL071W-syn-F | CAGCGGTAGTAACAAACGTCATGATGAC | PCRTag analysis |
| YEL070W-syn-F | TAGTAACCCTGCTATCCAGGATACCGTT | PCRTag analysis |
| YEL067C-syn-F | GACGGCGCTAACGAAGATAGTGCTACAT | PCRTag analysis |
| YEL065W-syn-F | CATCTTACCATTGGCCTGCATCCCATTG | PCRTag analysis |
| YEL064C-syn-F | TGAAAAGATGGCACGCAAAACATGTGGG | PCRTag analysis |
| YEL063C-syn-F | ACTCTGGGTAACGCTGTAAGCTAAGCTG | PCRTag analysis |
| YEL061C-syn-F | ACCATCGAAACCGGTGTTTGAGCTACCA | PCRTag analysis |
| YEL060C-syn-F | GCTAGTGCCGCTCAAGGTAGCAGTA | PCRTag analysis |
| YEL052W-syn-F | CGGCTTGAAGAGTGTTTTCTCAAGAGGT | PCRTag analysis |
| YEL050C-syn-F | TAACAAAGCAATGTGGCTACTACGGCCA | PCRTag analysis |
| YEL047C-syn-F | CTTCCATGAGCTTGATCTATCGTTAGGG | PCRTag analysis |
| YEL044W-syn-F | CGGTTTCCCAAGCCGTTTTAAAAGCGCT | PCRTag analysis |
| YEL043W-syn-F | TCACGCTAGCAGCCCTCCATTTAATAGT | PCRTag analysis |
| YEL042W-syn-F | CAGTGTCGGCGCTGCCAATAGTTTG | PCRTag analysis |
| YEL041W-syn-F | CGGTAGTACCGCTTATAGCTTATCAGCT | PCRTag analysis |
| YEL040W-syn-F | AAGCACCGCTACTAGCAGTAGTAAGACC | PCRTag analysis |
| YEL038W-syn-F | TCCAGCTCACGACAGCTTAGACTTAAAC | PCRTag analysis |
| YEL037C-syn-F | GGTCAAACCGATACTGCCAGGTGGA | PCRTag analysis |
| YEL030W-syn-F | TGCTCGTATCACTGCTAGCGACATCAGC | PCRTag analysis |
| YEL029C-syn-F | GAATGGAACACGATAGACGATTGGAGTC | PCRTag analysis |
| YEL022W-syn-F | CCACAGCGAGAAATCAACCAACGGTGCT | PCRTag analysis |
| YEL017W-syn-F | CTCAACCGACAATGATAGCAGTACTAGC | PCRTag analysis |
| YEL015W-syn-F | AGTCTTGACCGTCGCTAGCCAAAGTGGT | PCRTag analysis |
| YEL013W-syn-F | TAGCTTGTTAAGCAGTACTGACCCAGAC | PCRTag analysis |
| YER010C-syn-F | TCTACCGAAAACAACGGTACCGTTGCTC | PCRTag analysis |
| YEL011W-syn-F | CTACGAAGCTCATGTTGGCATCAGCTCA | PCRTag analysis |
| YER016W-syn-F | CAGCAGCTTAGGTATCAATGGCTCACGT | PCRTag analysis |
| YER020W-syn-F | TCAACCTAGCTTAAGCGATGCCAGCTCA | PCRTag analysis |
| YER026C-syn-F | TGATTTGCTGATCATACCGCAACCGTGA | PCRTag analysis |
| YER027C-syn-F | TGAGCTTGATGAGGCGTCAATGTCGTCG | PCRTag analysis |
| YER028C-syn-F | AGCCAAGCTTGAACCACTGCTGTGAACG | PCRTag analysis |
| YER032W-syn-F | ACGTAGCCCATTGAGATTTACCAGCAGC | PCRTag analysis |
| YER033C-syn-F | GTTTTTACTACCACCTCTCAATGGGCTC | PCRTag analysis |
| YER041W-syn-F | CGGTCCTTCAAGCATCACCAGTCATTCA | PCRTag analysis |
| YER042W-syn-F | CGGTGAAGAGAGCAAGAAAGACTCACCA | PCRTag analysis |
| YER045C-syn-F | ACTGTCACCGCTAGGAATGCTTGGG | PCRTag analysis |
| YER047C-syn-F | TGAACTTAAACTGCTCCACTGGACCAAG | PCRTag analysis |
| YER048C-syn-F | ATCGTGTTTGACCATACCGCCGTCGGTA | PCRTag analysis |
| YER051W-syn-F | CTCAGGTGTTTACGTTCCAAACGTTGGT | PCRTag analysis |
| YER053C-syn-F | ACGCTTGCTAGCAACGCTCATACTCTCA | PCRTag analysis |
| YER054C-syn-F | GCTCTTGCTAGGAGGCATATCAGTTCTA | PCRTag analysis |
| YER055C-syn-F | GCAGCTAGCCTCAACACTGCCTGAA | PCRTag analysis |
| YER056C-syn-F | AAAAGCCAAGCCGGCGACCAAACTAAAG | PCRTag analysis |
| YER059W-syn-F | GATCTTGGATGGCGATACCTCAAATAGC | PCRTag analysis |
| YER060W-syn-F | TTTCAGCGTCTTTGGCGCTGAGTTAGGT | PCRTag analysis |
| YER060W-syn-F | CTTCTTCAGTGTTGTTGCTGGCTTGGCT | PCRTag analysis |
| YER061C-syn-F | ACTACGACCAGGCAACAAAGCGCTAGCA | PCRTag analysis |
| YER062C-syn-F | GTCTCTAGTGCCACTGGTAGCAACG | PCRTag analysis |
| YER064C-syn-F | GTCAGCGCTGGTTGAGCTTCTGGTA | PCRTag analysis |
| YER065C-syn-F | TCTGCTAAAATTGTGAACGGCCAAGGCA | PCRTag analysis |
| YER066W-syn-F | CCATCGTGCTTTGGTTGGCTTGTTAGGT | PCRTag analysis |
| YER069W-syn-F | CGATACCGCCAGCACTTTGAATAGCAGC | PCRTag analysis |
| YER070W-syn-F | GCGTCCAGGCGCTTTCGCTTTGTATTTA | PCRTag analysis |
| YER073W-syn-F | CGGTGGTGCTCGTCACGGTTCA | PCRTag analysis |
| YER075C-syn-F | ACTGTCGCTTTCCAATGACAACATGCTC | PCRTag analysis |
| YER076C-syn-F | GTCGCATGATGAATTGGTGCTCCAGGTT | PCRTag analysis |
| YER081W-syn-F | CGATTTGGATTATGCCACTTCACGTGGC | PCRTag analysis |
| YER086W-syn-F | TAGCCAAGGCGTTGGCTTAAGCAGTAGA | PCRTag analysis |
| YER087W-syn-F | TCAATGGCTACCTTTGGGTTTGCGTAGC | PCRTag analysis |
| YER089C-syn-F | ATCAGCGAAGCTTCTAACGCTGGTTCTA | PCRTag analysis |
| YER091C-syn-F | GATAGGAGGACGAACGTAACGACTGCCA | PCRTag analysis |
| YER096W-syn-F | CAACCCTTCAGGTAGCGGTAGCAGCAAT | PCRTag analysis |
| YER098W-syn-F | TGATCCAAGTATCGCTAAGAGCCCTTCA | PCRTag analysis |
| YER099C-syn-F | GACGTCGCCAACCAATAACATTCTGCTG | PCRTag analysis |
| YER101C-syn-F | GCTATGTGATAAAATGTCGTAGCCGCCA | PCRTag analysis |
| YER103W-syn-F | CGAAAGAGCTAAACGTACCTTAAGCAGC | PCRTag analysis |
| YER105C-syn-F | TGGAACTGAACCTGATTCACGGCTTGAC | PCRTag analysis |
| YER107C-syn-F | TGACAAGGCATAAGCGAAAACTGAGCCG | PCRTag analysis |
| YER109C-syn-F | AGCGATTGACAAAGGACTGCTAGCGCTG | PCRTag analysis |
| YER110C-syn-F | ATCGTCAATATGACCGCTCAAGCTGCTA | PCRTag analysis |
| YER113C-syn-F | ACCAACGCTGTTAGCCAATGACATCCAG | PCRTag analysis |
| YER114C-syn-F | ACCACTGGCCAACAAAAATGGTGAACTG | PCRTag analysis |
| YER115C-syn-F | ATCGCATGAGAATCTGGCCAAAGGTGAA | PCRTag analysis |
| YER116C-syn-F | CTTTGAACGGCACAAGGCACAGTGACCA | PCRTag analysis |
| YER118C-syn-F | ACTCAACAAAATAACGCCAGCGCTGGCG | PCRTag analysis |
| YER119C-syn-F | AAAGCTGATGCTGGTACTACCGGTAGCA | PCRTag analysis |
| YER122C-syn-F | ACGTGAGGCGGTCAAAATACTTGAACGG | PCRTag analysis |
| YER129W-syn-F | ATTGGCCGCCTCATCAACCAACTCAAGT | PCRTag analysis |
| YER130C-syn-F | AACAACCAAACCGTGGCTGCTGCTACTT | PCRTag analysis |
| YER132C-syn-F | TGATGAACGACGGCTTGGAGCCTTAGGA | PCRTag analysis |
| YER141W-syn-F | TAGACCTTTCAGCTTGAGTAGTCCAGTC | PCRTag analysis |
| YER143W-syn-F | CAGCAGCGACAAACCATTGACCCCAACT | PCRTag analysis |
| YER144C-syn-F | AGTGCTTGGGGTGTTCAACTTGCTCAAG | PCRTag analysis |
| YER151C-syn-F | GCAACTGCTGGTACCAACACGGCTGTTA | PCRTag analysis |
| YER152C-syn-F | ACTACGTAAGTGAGCGATACAACGCTGA | PCRTag analysis |
| YER161C-syn-F | TGGGCCGTTTGAATGCTTGCTCTTTGAA | PCRTag analysis |
| YER163C-syn-F | GCTGGTCAACAAAACTCTCTTGCCGCTC | PCRTag analysis |
| YER170W-syn-F | TCGTTACGTCCATGTCCCATCAGGTCGT | PCRTag analysis |
| YER174C-syn-F | GTCGTCACTGCTACCTGATGATTCCTCG | PCRTag analysis |
| YER175C-syn-F | CAATTCTGGTCTTCTTCTCAAGCTCTCC | PCRTag analysis |
| YER177W-syn-F | AAATGTCATCGGCGCCAGAAGAGCTAGC | PCRTag analysis |
| YER178W-syn-F | TGCCAGCCGTAGTAGTGCTATGACC | PCRTag analysis |
| YER180C-syn-F | TCTTTCAAAGACTGGGTAGGTACCGGCG | PCRTag analysis |
| YER182W-syn-F | CGGTGGTTCATTCTTGGGTGGTTGGTAT | PCRTag analysis |
| YER184C-syn-F | GTGTGAGGCGCTTGAGGCATCCAAGAAT | PCRTag analysis |
| YER188W-syn-F | TCGTAGAAGCGACGCTTTGGGTGTTACC | PCRTag analysis |
| YEL071W-syn-R | ATTCAAACGCTCGGTAACGGCAGCGCTA | PCRTag analysis |
| YEL070W-syn-R | ATGTGGGTCCTTGCCACCTTTGACAGCA | PCRTag analysis |
| YEL067C-syn-R | TTGCAGCAGAAGATTCCCACCAGACAAT | PCRTag analysis |
| YEL065W-syn-R | AACAACGACCCAGCCGATAACTTCTGGA | PCRTag analysis |
| YEL064C-syn-R | TGCTTTGGGTTTCATCGTCGACTGGACC | PCRTag analysis |
| YEL063C-syn-R | CCGTGTTAACGGTGAGGACACCTTTAGC | PCRTag analysis |
| YEL061C-syn-R | CGAAGTTGCTGGTCCTTTGTTTCAGGAC | PCRTag analysis |
| YEL060C-syn-R | TGGCGTTGCCAAGAACGCTAATGTCGTC | PCRTag analysis |
| YEL052W-syn-R | AGCAACATCGGTAACTTGGAACTCATCG | PCRTag analysis |
| YEL050C-syn-R | TAGCCCAGGCTTACGTTGGTATAGAAGT | PCRTag analysis |
| YEL047C-syn-R | CCAATTAGGCGGTCATAGCGTTGCTCGT | PCRTag analysis |
| YEL044W-syn-R | AGCACCACGTAACTTCAAATACTCCTGG | PCRTag analysis |
| YEL043W-syn-R | GTTGGCTGGGCTGCTAGCGCTTTTATGT | PCRTag analysis |
| YEL042W-syn-R | ACCCATAATACTGACGCCGTCGCCTTCA | PCRTag analysis |
| YEL041W-syn-R | AACGCTGTATGGTGAAGCGGTGATAACG | PCRTag analysis |
| YEL040W-syn-R | GCCAGCATTGTTGCCTGACATGCTTGAG | PCRTag analysis |
| YEL038W-syn-R | AACTGGAGCGTTGCCTGGTCTACTG | PCRTag analysis |
| YEL037C-syn-R | CGCCCCAGAAGGTAGCCAACCA | PCRTag analysis |
| YEL030W-syn-R | AACCAACTCTCTCTCGCCTTGAAAGACC | PCRTag analysis |
| YEL029C-syn-R | AGTCAGATGCATGGGTACCTATTATGCC | PCRTag analysis |
| YEL022W-syn-R | AGGATACAACTGTAATCTGTCGCCGCTG | PCRTag analysis |
| YEL017W-syn-R | GCTGCTGGTAGGCTGACCAGCA | PCRTag analysis |
| YEL015W-syn-R | GTCTTGTTGTGGCTGGCTTTGATGGGTA | PCRTag analysis |
| YEL013W-syn-R | AACTAAGTGTGGTAAACCGCCGGCTCTA | PCRTag analysis |
| YER010C-syn-R | GCGTCCAAAGACTACTGTCCCATTCGAT | PCRTag analysis |
| YEL011W-syn-R | TAAACCGTCCTCAACATTCTTGCTGGCG | PCRTag analysis |
| YER016W-syn-R | GCCGTTAACCTCACCGTTTGAATTAGCG | PCRTag analysis |
| YER020W-syn-R | GCTTGGTAAAGCCCATAAGGTGCTGATA | PCRTag analysis |
| YER026C-syn-R | CGGCAAGCCACACTATGTTCAGAGAGCT | PCRTag analysis |
| YER027C-syn-R | CAACGAGGCCAGTTTAGCTTATACCTTC | PCRTag analysis |
| YER028C-syn-R | AGTCGCCCAGAACTGTGACGATGTTAAC | PCRTag analysis |
| YER032W-syn-R | CAATGGCTTAAAAGGCTCGATGCTAGGG | PCRTag analysis |
| YER033C-syn-R | ACACAGCCAGCAGCCACATTACGCT | PCRTag analysis |
| YER041W-syn-R | ATGCATAATAGCAACGGTGCTAGGCCAA | PCRTag analysis |
| YER042W-syn-R | GTGGGCAAATAAACCTGATCTGTACTGG | PCRTag analysis |
| YER045C-syn-R | CCCTTCAGCTGCTATCTATCCTTCATTC | PCRTag analysis |
| YER047C-syn-R | TATGTTGTTGTTCGGTCCTCCTGGCACC | PCRTag analysis |
| YER048C-syn-R | CCACCCAGATAAACACCCAGACGATCCT | PCRTag analysis |
| YER051W-syn-R | GTTCTCCTCAGCTTTCTCACCGTTAACC | PCRTag analysis |
| YER053C-syn-R | TAAGCAGCAGACCACCATGCCACCATTC | PCRTag analysis |
| YER054C-syn-R | CAGATCAGGTAACGGCGTTCAAGCTCGT | PCRTag analysis |
| YER055C-syn-R | TTTCTTACCAGCCGCTGACATCCCTACC | PCRTag analysis |
| YER056C-syn-R | CGCTTATGAGAAGTGGAGCTGGGTTCCA | PCRTag analysis |
| YER059W-syn-R | TGGGGTGGTGGTAACTGAGTTACAACGT | PCRTag analysis |
| YER060W-syn-R | AGTGTAATCGGCAGCGTAGGTGGTCCAA | PCRTag analysis |
| YER060W-syn-R | GGCAACGGTATACATACCTGGAACGTTG | PCRTag analysis |
| YER061C-syn-R | AACCGCTTGTGCTACCGGCAACAATAGT | PCRTag analysis |
| YER062C-syn-R | TGACGCCGAACATGTCATTCAAGTTAGC | PCRTag analysis |
| YER064C-syn-R | CGGTTTCGACGCTAGCTTAGCTCCTATT | PCRTag analysis |
| YER065C-syn-R | TATGAGAGCTAGAGCTTTCGCCCCTTAC | PCRTag analysis |
| YER066W-syn-R | GGTGTCGCTTCTAACCAATAAACCTGAG | PCRTag analysis |
| YER069W-syn-R | TGGCAAAGCCATGACCCAGAAATCAACG | PCRTag analysis |
| YER070W-syn-R | CTTCTCATATCTAGTGTACAAGGCCTCG | PCRTag analysis |
| YER073W-syn-R | GATGCCGCTCTGACCAAAACCGCCAAAT | PCRTag analysis |
| YER075C-syn-R | TAGTGTCATCCCAGAATGGTTTCAGCAC | PCRTag analysis |
| YER076C-syn-R | CGGCGAAAGCACTTTGTGCCGTGCTAAA | PCRTag analysis |
| YER081W-syn-R | GTCTTTCATGGCGGCGAATTGAGGAGCT | PCRTag analysis |
| YER086W-syn-R | TCTCTCTTCGGCTAACTTAGCGCATTCG | PCRTag analysis |
| YER087W-syn-R | GTCAGCCCAAGCTGAGACGAATGGAATC | PCRTag analysis |
| YER089C-syn-R | CCCAGATATCTTGGAGCACAGCTTGGAC | PCRTag analysis |
| YER091C-syn-R | TCCTACCACCACCATCGGCAGTTTTCCA | PCRTag analysis |
| YER096W-syn-R | ATAAGCATCGCCCAACAAATACTGAGCG | PCRTag analysis |
| YER098W-syn-R | AGCTCTGTCTTCGTAGGTGGTGCTACTG | PCRTag analysis |
| YER099C-syn-R | CGCTAACTTGTTAGAAACCGCTGGCTGC | PCRTag analysis |
| YER101C-syn-R | CGGCGCTGCTGTTTTAAGCGAACACTTT | PCRTag analysis |
| YER103W-syn-R | GTAGGCGACAGCCTCGTCTGGATTGATA | PCRTag analysis |
| YER105C-syn-R | CCCACAAGGCTATGCTAACGTTTTCGCT | PCRTag analysis |
| YER107C-syn-R | TAACCCAAACAGAGCTCCAGGTAGTAAC | PCRTag analysis |
| YER109C-syn-R | AGCCCCACCAACCAAGACCGCT | PCRTag analysis |
| YER110C-syn-R | TTGGAATGCTATCGACGAAAGTACCCGT | PCRTag analysis |
| YER113C-syn-R | CCACTGCCCAGGTGCTAGCAAAAATTAT | PCRTag analysis |
| YER114C-syn-R | CACCCCAACCGTCAGTTTATCAAAGGCT | PCRTag analysis |
| YER115C-syn-R | TGCTTGCGATCCTAACAAACAGCAGAAC | PCRTag analysis |
| YER116C-syn-R | CATGCACACTGAAGAGCCTGAAGCTTCA | PCRTag analysis |
| YER118C-syn-R | ATTTCCTAGATTCACCTGGTGGGGCATC | PCRTag analysis |
| YER119C-syn-R | CGCCACTGAATCAAGCCCATTAATCAGA | PCRTag analysis |
| YER122C-syn-R | AAGCGATAGCCCATTAGATACTGACAGC | PCRTag analysis |
| YER129W-syn-R | TGGAACATGATCGAAGTCCCAGCTGACG | PCRTag analysis |
| YER130C-syn-R | CCAGACTGAAAATAGCAGTAGCCAGAAG | PCRTag analysis |
| YER132C-syn-R | CAGACGTGCTAGCCATCCATTGCAAAGC | PCRTag analysis |
| YER141W-syn-R | ACCAATGGCTCTGCCCCATAAACGG | PCRTag analysis |
| YER143W-syn-R | AAACAACAAACTAGCGGCGAATTCAGCG | PCRTag analysis |
| YER144C-syn-R | CAGCGTTATCAAGCCATTATCAGGTACC | PCRTag analysis |
| YER151C-syn-R | CATCGAGCCTTTAGGCAGCATCGCTTTG | PCRTag analysis |
| YER152C-syn-R | CTATAGCTTGGAGACTCGTAGACGTTTG | PCRTag analysis |
| YER161C-syn-R | AAGAAGCATCGGTGCTTCACACGCTCCT | PCRTag analysis |
| YER163C-syn-R | AGCTAATCCTGGTCGTGTTGCTACCTTG | PCRTag analysis |
| YER170W-syn-R | GGTTTCACCGCTAACAGTGCCGAAGATA | PCRTag analysis |
| YER174C-syn-R | CCCATGTAAGACCATGTCACAGGTTTTG | PCRTag analysis |
| YER175C-syn-R | CTGGGGCTACGCTGATCCTATCTTTCCA | PCRTag analysis |
| YER177W-syn-R | GGTAGCTTTTTCACGGGCGTCACCTGAT | PCRTag analysis |
| YER178W-syn-R | ATCATAGGCCTTAACTTCGGCTTCGGTA | PCRTag analysis |
| YER180C-syn-R | TTTGGATTACATCCCAGACAGTCCTAGC | PCRTag analysis |
| YER182W-syn-R | AACACCGCCAGGGGTGTTCAAAGCTCTA | PCRTag analysis |
| YER184C-syn-R | TGCTAGCTTGCAGAAGGGTTTGGCTAAT | PCRTag analysis |
| YER188W-syn-R | GTTCCAACACAACATCTGACCAGGTCTG | PCRTag analysis |
| YER162C-syn-F | AGTGATTTCGCCTGAAGCTGAAGCCAAA | PCRTag analysis |
| YER162C-syn-R | CGAATCAGAAGGTATCCCTGACAGCGTT | PCRTag analysis |
| YER163C-syn-F | GCTGGTCAACAAAACTCTCTTGCCGCTC | PCRTag analysis |
| YER163C-syn-R | AGCTAATCCTGGTCGTGTTGCTACCTTG | PCRTag analysis |
| YER164W-syn-F | TGCTAAGAAAAGCGCTTCAAGTAGCGAC | PCRTag analysis |
| YER164W-syn-R | ACTTGGGCCGGTTGGCAACTTCTTGCTA | PCRTag analysis |
| YER161C-wt-F | GGGACCATTGCTGTGTTTCGATTTGCTG | PCRTag analysis |
| YER161C-wt-R | GAGGTCTATTGGAGCAAGTCATGCGCCA | PCRTag analysis |
| YER162C-wt-F | TGGCTCTGAAGGTTCACTATACGGCCTT | PCRTag analysis |
| YER162C-wt-R | GGAACATTTGCTTGGTGCTTTGGAGAGT | PCRTag analysis |
| YER163C-wt-F | CGAAGTAAGTAGTACCCGTTTACCCGAT | PCRTag analysis |
| YER163C-wt-R | TGCAAACCCAGGAAGAGTGGCCACACTA | PCRTag analysis |
| YER164W-wt-F | TGATAGCCAACGTGCTTCGTTGGAAGAC | PCRTag analysis |
| YER164W-wt-R | TCCCAGGCCCATCTCATCTGCCAGTATA | PCRTag analysis |
| URA-F | CTTAACTATGCGGCATCAGAGC | Amplification of *URA3* marker |
| URA-R | CCTGATGCGGTATTTTCTCCTTACG | Amplification of *URA3* marker |
| ura-test-R-1 | CTAACTCCAGTAATTCCTTGGTGGT | Verification for *URA3* marker |
| ura-tesr-F-1 | TCAACAGTATAGAACCGTGGATGATG | Verification for *URA3* marker |
| D-YER160CF-F | ACAGTGGGCTAAGAATATACAACCG | Deletion of YER161C |
| D-YER160CF-R | TCTGATGCCGCATAGTTAAGAGAAGGGCATACGCTAAGGAATATTG | Deletion of YER161C |
| D-YER162CR-F | GGAGAAAATACCGCATCAGGATTTCAGTCTGATTCCTCTGACATCTC | Deletion of YER161C |
| D-YER162CR-R | TGGAATTTGCACCTGCTGTAAC | Deletion of YER161C |
| DH-YER162C-F | ATTTCAGTCTGATTCCTCTGACATCTC | Deletion of YER161C |
| DH-YER160C-R | CAGAGGAATCAGACTGAAATAGAAGGGCATACGCTAAGGAATATTG | Deletion of YER161C |
| test-YER161C-F | TGACGTATTGTCATACTGACGTATCTC | Verification for YER161C deleted |
| test-YER161C-R | GAAGACACAGAATTATACATCCCTCC | Verification for YER161C deleted |
| D-YER161CF-F | AAGATACTCCACGCAATGTTGC | Deletion of YER162C |
| D-YER161CF-R | TCTGATGCCGCATAGTTAAGGAGGCTGAAACGGTTTGAATAATTAGG | Deletion of YER162C |
| D-YER163CR-F | GGAGAAAATACCGCATCAGGCATTCACCTGTATTTATTTACAGTCTCC | Deletion of YER162C |
| D-YER163CR-R | TAGCTATGAGTTTAGTCTCGAGGTG | Deletion of YER162C |
| DH-YER161C-R | GGAGGCTGAAACGGTTTGAATAATTAGG | Deletion of YER162C |
| DH-YER163C-F | ATTCAAACCGTTTCAGCCTCCATTCACCTGTATTTATTTACAGTCTCC | Deletion of YER162C |
| test-YER162-R | TTGTCACCAACACCTAGCAGAC | Verification for YER162C deleted |
| test-YER162C-F | ACCAAGCTTCCTCACGTTTATCC | Verification for YER162C deleted |
| D-YER162CF-F | TGGAATGAAGTCTGACCTGTTCAATG | Deletion of YER163C |
| D-YER162CF-R | GGAGAAAATACCGCATCAGGTGATTAACTATGTAAGGTTCTCCTCTTGC | Deletion of YER163C |
| D-YER164WR-F | TCTGATGCCGCATAGTTAAGTTGGCCATACAACTTTTTATAAGCCC | Deletion of YER163C |
| D-YER164WR-R | TGATCTTTTACCGTATATGGGGCAG | Deletion of YER163C |
| DH-YER162C-R | TGATTAACTATGTAAGGTTCTCCTCTTGC | Deletion of YER163C |
| DH-YER164W-F | GAACCTTACATAGTTAATCATTGGCCATACAACTTTTTATAAGCCC | Deletion of YER163C |
| test-YER163C-F | GTTCATCCATTGGGCATATCTTCG | Verification for YER163C deleted |
| test-YER163C-R | GTGGCCTACCAGTGATATCCAAC | Verification for YER163C deleted |
| D-YER163CF-F | ACCCAGATACCACTGTTGTCATTAG | Deletion of YER164W |
| D-YER163CF-R | GGAGAAAATACCGCATCAGGAGGTTCTGCTTTGAATTGGGTATAGG | Deletion of YER164W |
| D-YER165W-R | CTTCGGAAACAGAAGGTTCTAAGTC | Deletion of YER164W |
| H-165-F | AATTTTTCTTCACCACATTTTCCATTGT | Deletion of YER164W |
| H-163-R | AAATGTGGTGAAGAAAAATTAGGTTCTGCTTTGAATTGGGTATAGG | Deletion of YER164W |
| H-60-64-R | AAATGTGGTGAAGAAAAATTAGAAGGGCATACGCTAAGGAATATTG | Deletion of YER164W |
| D-YER161-164R-F | GGAGAAAATACCGCATCAGGAATTTTTCTTCACCACATTTTCCATTGT | Deletion of YER161C-164W |
| test-YER164W-F | ATATCACTGGTAGGCCACTTCTTC | Verification for YER164W deleted |
| test-YER164W-R | TGTCATACAAAGCCTTGTTGTC | Verification for YER164W deleted |
